# Fast Switching of Bright Whiteness in Channeled Hydrogel Networks

**DOI:** 10.1002/adfm.202000754

**Published:** 2020-05-28

**Authors:** Amanda Eklund, Hang Zhang, Hao Zeng, Arri Priimagi, Olli Ikkala

**Affiliations:** ^1^ Department of Applied Physics Aalto University P.O. Box 15100 Espoo FI 02150 Finland; ^2^ Smart Photonic Materials Faculty of Engineering and Natural Sciences Tampere University P.O. Box 541 Tampere FI‐33101 Finland

**Keywords:** double networks, hydrogels, interpenetrating networks, lower critical solution temperature, whiteness

## Abstract

Beside pigment absorption and reflection by periodic photonic structures, natural species often use light scattering to achieve whiteness. Synthetic hydrogels offer opportunities in stimuli‐responsive materials and devices; however, they are not conventionally considered as ideal materials to achieve high whiteness by scattering due to the ill‐defined porosities and the low refractive index contrast between the polymer and water. Herein, a poly(*N*‐isopropylacrylamide) hydrogel network with percolated empty channels (ch‐PNIPAm) is demonstrated to possess switchable bright whiteness upon temperature changes, obtained by removing the physical agarose gel in a semi‐interpenetrating network of agarose and PNIPAm. The hydrogel is highly transparent at room temperature and becomes brightly white above 35 °C. Compared to conventional PNIPAm, the ch‐PNIPAm hydrogel exhibits 80% higher reflectance at 800 nm and 18 times faster phase transition kinetics. The nanoscopic channels in the ch‐PNIPAm facilitate water diffusion upon phase transition, thus enabling the formation of smaller pores and enhanced whiteness in the gel. Furthermore, fast photothermally triggered response down to tens of milliseconds can be achieved. This unique property of the ch‐PNIPAm hydrogel to efficiently scatter visible light can be potentially used for, e.g., smart windows, optical switches, and, as demonstrated in this report, thermoresponsive color displays.

## Graphical abstract

A channeled poly(*N*‐isopropylacrylamide) hydrogel is prepared using agarose as the removable template. The hydrogel shows bright whiteness and 18 times faster kinetics of transparency change compared to standard poly(*N*‐isopropylacrylamide) hydrogels. The highly efficient scattering of visible light can be utilized for applications such as smart windows and switchable passive displays.

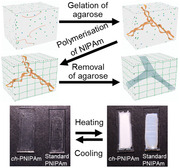

## 1 Introduction

White coloration is ubiquitous in nature, as widely observed in different species for camouflage or signaling.^[^ ^1^, ^2^, ^3^, ^4^, ^5^, ^6^, ^7^, ^8^, ^9^ ^]^ Whiteness is a result of uniform spectral reflectance across the visible range, mainly achieved by multiple scattering through randomly constructed skin architectures. For instance, the white beetles in the genus *Cyphochilus* have anisotropic intrascale chitin networks as dense scattering media,^[^ ^2^ ^]^ while the white spots in the skin of cuttlefish *Sepia officinalis* contain randomly ordered proteinaceous microspheres.^[^ ^4^ ^]^ In industry, titanium dioxide nanoparticles and cellulose fibers have been commonly used to achieve high whiteness in personal‐care products, paints, paper, and markers.^[^ ^9^ ^]^ While natural systems often adopt a specifically evolved strategy to pack their random filaments, optimize the scattering mean‐free path, and hence increase the scattering efficiency,^[^ ^2^ ^]^ artificial systems often seek for materials with high refractive indices to enhance the contrast between scattering centers and surrounding medium.

Normally, the appearance of whiteness is static and cannot be easily changed after fabrication or formation. To realize a system that can change its color, transparency, or whiteness, implementation of artificial switchable materials is favorable. For this, the phase transition of liquid crystals,^[^ ^8^, ^10^, ^11^ ^]^ or deformation‐induced air pockets inside a liquid‐infused elastomer,^[^ ^7^ ^]^ have been utilized. However, these systems rely on a large refractive index contrast between the synthetic material and air, or molecular orientation controlled by electrical field/temperature, which either has limited capacity in whiteness change or is not suitable for an aqueous environment.

Hydrogels are cross‐linked polymeric networks swollen with water and have attracted immense research interest due to their stimuli‐responsiveness, flexibility and softness, potential for biomedical applications, etc.^[^ ^12^, ^13^, ^14^, ^15^, ^16^ ^]^ However, they are normally not considered as ideal systems to achieve high whiteness due to the low refractive contrast between the polymer network and the surrounding water. Common hydrophilic polymers have a refractive index between 1.40 and 1.60 in the visible range,^[^ ^17^ ^]^ while the refractive index of water is 1.33. This results in the highly transparent appearance of hydrogel with water content typically more than 90 wt%. For hydrogels like poly(*N*‐isopropylacrylamide) (PNIPAm), some degree of whiteness can be achieved by heating the sample above its lower critical solution temperature (LCST) to induce the phase separation.^[^ ^18^ ^]^ This leads to the formation of polymer‐rich and polymer‐poor microphases, forming light‐scattering centers.^[^ ^19^, ^20^, ^21^ ^]^ However, due to the intrinsic low refractive index contrast and the ill‐defined size distribution of the pores, strong and bright whiteness in hydrogel systems has not been reported, except for composite hydrogels containing light‐scattering particles that lack switchability.^[^ ^22^ ^]^ Different types of interpenetrating networks or porous hydrogels based on PNIPAm have been reported, such as using aluminum alginate,^[^ ^23^, ^24^, ^25^ ^]^ silk fibroin,^[^ ^26^, ^27^ ^]^ cellulose,^[^ ^28^, ^29^ ^]^ silica nanoparticles,^[^ ^30^ ^]^ or microemulsions.^[^ ^31^ ^]^ These hydrogels possess improved mechanical properties and/or phase transition kinetics. However, they do not exhibit particularly interesting optical properties.

In this work, we demonstrate a switchable channeled PNIPAm (ch‐PNIPAm) hydrogel that shows bright whiteness when heated above its phase transition temperature. The ch‐PNIPAm hydrogel is obtained by forming a semi‐interpenetrating network of physically crosslinked agarose and chemically crosslinked PNIPAm networks, after which the agarose network is removed to form emptied water‐transporting channels. The ch‐PNIPAm hydrogel shows high reflectance (>70%) across 400–700 nm at a thickness of 0.95 mm and a polymer content around 10 wt%. The whiteness is significantly enhanced especially in the long‐wavelength range (600–800 nm) compared to the standard PNIPAm hydrogel. The kinetics of the phase transition is characterized by the transmittance change upon local photothermal heating in the samples of millimeter thickness, which shows a macroscopic response time down to tens of milliseconds, 18 times faster than standard PNIPAm. The bright whiteness of the hydrogel and the fast kinetics can be attributed to the modification of the microscopic porosity distribution of the PNIPAm hydrogel and the formation of microscopic water‐transporting channels by removal of the agarose template. The principle can also be applied to other LCST hydrogels, and the high whiteness can be potentially utilized for various optical applications, such as smart windows, reflection screens, and flexible passive displays.

## 2 Results and Discussion

### 2.1 Synthesis and Spectral Properties of the ch‐PNIPAm Hydrogel

**Figure 1:**
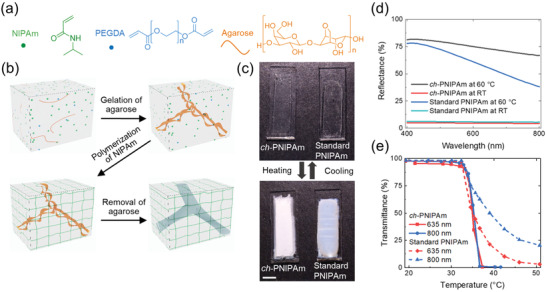
Synthesis and characterization of the channeled‐PNIPAm (ch‐PNIPAm) hydrogel. a) Chemical structures of *N*‐isopropylacrylamide (NIPAm), poly(ethylene glycol) diacrylate (PEGDA), and agarose. b) Schematic illustration of the synthesis of the hydrogel. c) Photographs of the ch‐PNIPAm hydrogel and standard PNIPAm hydrogel at room temperature and at 60 °C. Scale bar: 5 mm. As‐prepared thickness: 1 mm. d) Total reflectance of the ch‐PNIPAm hydrogel and standard PNIPAm hydrogel films at 60 °C and room temperature. Film thickness: 2.7 mm (ch‐PNIPAm) and 2.6 mm (standard PNIPAm) at room temperature, 0.95 mm (ch‐PNIPAm) and 0.86 mm (standard PNIPAm) at 60 °C. e) Transmittance of the ch‐PNIPAm and standard PNIPAm hydrogels as a function of temperature at different wavelengths. Film thickness: 2.7 mm (ch‐PNIPAm) and 2.6 mm (standard PNIPAm) at room temperature. Solid and dashed lines are only to guide the eye.

The chemical structures of the monomer (NIPAm), cross‐linker poly(ethylene glycol) diacrylate (PEGDA, *M_n_* = 10000) and agarose are presented in **Figure** **1**a, and the synthesis of the hydrogel is schematically shown in Figure 1b. Briefly, all the components are first dissolved in water to form a homogeneous solution with 10 wt% NIPAm, 0.3 wt% agarose, and relative to NIPAm, 0.05 mol% PEGDA, and 1 mol% photoinitiator Irgacure 2959. The first network of agarose is formed by gelation at 4 °C, which typically shows a fibril diameter around 10 nm with a mesh size in the range of 10–1000 nm.^[^ ^32^, ^33^ ^]^ The second network of PNIPAm is formed within the agarose network using UV‐initiated free radical polymerization at room temperature. Finally, the agarose is removed by heating the sample above the melting point (*T*_m_ ≈ 32 °C)^[^ ^34^ ^]^ of the agarose and repeatedly washing the gel in alternating cold and hot water baths, which induces the swelling and shrinking of PNIPAm network for efficient removal of the agarose. FTIR confirmed the complete removal of the agarose, as shown in Figure S1 in the Supporting Information. The removal of agarose is critical for achieving high whiteness, as shown in Figure S2 in the Supporting Information, and also ensures the consistency of the results, as the agarose melts above the LCST of the PNIPAm and may cause heterogeneity inside the gel. The as‐prepared hydrogel contains a PNIPAm network channeled by the voids formed through the removal of the agarose network, thus denoted as channeled‐PNIPAm (ch‐PNIPAm). The ch‐PNIPAm is highly transparent at room temperature and becomes brightly white when heated above its LCST, as shown in Figure 1c.

To quantify the whiteness of the hydrogel, the total reflectance of ch‐PNIPAm films was measured using an integrating sphere at different wavelengths. Standard PNIPAm gel films were prepared using the same composition but without the agarose, and the films were also treated with the same washing cycles as the ch‐PNIPAm. The data are plotted in Figure 1d and the measurement details are given in the Experimental Section. The films have an as‐prepared thickness of 1 mm and a swollen thickness of 2.7 and 2.6 mm for ch‐PNIPAm and standard PNIPAm, respectively. Above the LCST, the film thicknesses decrease to 0.95 ± 0.07 mm and 0.86 ± 0.09 mm for ch‐PNIPAm and standard PNIPAm, respectively. For a summary of the film thicknesses before and after swelling/shrinking, see Figure S3 in the Supporting Information. The higher thickness of the shrunken ch‐PNIPAm may be attributed to the presence of porous channels, which are preserved, at least partly, in the shrunken ch‐PNIPAm due to the chemical cross‐linking. This leads to a larger fractional volume of voids and thus a higher shrunken thickness. It should be noted that samples with the same as‐prepared thickness are compared despite the slight difference in the shrunken thickness, i.e., water content. This is important to ensure the experimental consistency, as the samples are prepared with the same PNIPAm/PEGDA composition, apart from the porous channels in ch‐PNIPAm that contain purely water.

The hydrogel films were covalently attached to glass substrates to ensure the flatness and easy handling. At room temperature, the ch‐PNIPAm and standard PNIPAm gels are highly transparent, having a total reflectance of around 5% and 6%, respectively. When heated to 60 °C, both samples show a drastic increase in reflectance as a result of the phase separation inside the gel. However, the reflectance of standard PNIPAm hydrogel is not uniform as seen from the significant decrease in reflectance as the wavelength increases from 400 to 800 nm. In contrast, the ch‐PNIPAm hydrogel exhibits a much more enhanced reflectance compared to standard PNIPAm hydrogel, as evidenced by the 67% reflectance at 800 nm in the ch‐PNIPAm hydrogel compared to only 38% in the standard PNIPAm hydrogel. This endows the ch‐PNIPAm hydrogel with an appearance of bright whiteness above LCST, in contrast to the standard PNIPAm (Figure 1c). The high reflectance is remarkable considering that the shrunken thickness of the ch‐PNIPAm hydrogel film is 0.95 mm and the polymer content is only 10 wt%. It should be noted that an even higher reflectance can be achieved in the ch‐PNIPAm hydrogel by using *N*,*N′*‐methylenebisacrylamide (BIS) as the crosslinker instead of PEGDA, as shown in Figure S4 in the Supporting Information. However, such a sample has very weak mechanical stability and breaks apart easily during handling.

Beyond the visible range, the total reflectance of the ch‐PNIPAm hydrogel shows a small decrease in the near infrared region (800–1000 nm) and a significant drop below 350 nm, as shown in Figure S5 in the Supporting Information. To further quantify the whiteness of the hydrogels, the CIELAB color spaces, as specified by the International Commission on Illumination, have been measured and summarized in **Table** **1**.^[^ ^22^, ^35^ ^]^ As the reference, a grade 1 filter paper was used. The high whiteness of the ch‐PNIPAm can be seen from the higher L* value and smaller a* and b* values compared to standard PNIPAm. The lightness of the ch‐PNIPAm gel is as high as the filter paper, while the color is slightly bluer due to the lower reflectance in the red part of the spectrum.

**Table 1:** CIELAB color space of hydrogel samples

Sample	L* (lightness)	a* (red‐green)	b* (yellow‐blue)
standard PNIPAm	82.79	−2.71	−8.68
ch‐PNIPAm	90.86	−1.12	−2.87
Reference (filter paper)	90.99	−0.20	−0.58

The enhanced whiteness in the ch‐PNIPAm hydrogel can be compared to another natural system in water, i.e., the white spots on the skin of cuttlefish *Sepia officinalis*, which show a reflectance between 30% to 70% in the visible range as the thickness varies between 0.1 and 0.3 mm.^[^ ^4^ ^]^ Though the cuttlefish can achieve equivalent whiteness at a smaller thickness compared to the ch‐PNIPAm hydrogel, it should be noted that the leucophores, which are responsible for the scattering of white light in the case of cuttlefish, do not have the ability to change their optical properties such as transparency. The cuttlefish uses the active chromatophores on top of the leucophores to vary its skin pattern.^[^ ^4^ ^]^

The ch‐PNIPAm hydrogel also demonstrates a sharper phase transition in its transparency and more efficient attenuation of transmitted light compared to the standard PNIPAm hydrogel, which is characterized by the equilibrium transmittance of hydrogel films as shown in Figure 1e. The abrupt change in the transmittance in ch‐PNIPAm hydrogel takes place between 33 and 36.7 °C, where it drops from around 95% to below 1% for both 635 and 800 nm. In contrast, the phase transition of the standard PNIPAm hydrogel shows a gradual decrease in transmittance starting from 33 °C, which remains above 3% and 20% even at 50 °C for 635 and 800 nm, respectively. While it has been reported that sharp transitions in PNIPAm hydrogels can be achieved also by introducing charged groups in the hydrogel, for example by copolymerization with acrylic acid,^[^ ^36^, ^37^ ^]^ the present ch‐PNIPAm hydrogel shows that this can also be achieved by simply modifying the physical structure. It should be noted that here the sharper transition refers to the transparency, not the volume. In principle, pure structural change in the ch‐PNIPAm compared to the standard PNIPAm should not affect the equilibrium thermodynamics. The high whiteness and sharp transition are favorable properties to be utilized as optical switch in aqueous environments due to the strong light attenuation and the sharp transition between transparent and white states.

### 2.2 Influence of the Composition of the ch‐PNIPAm Hydrogel

**Figure 2:**
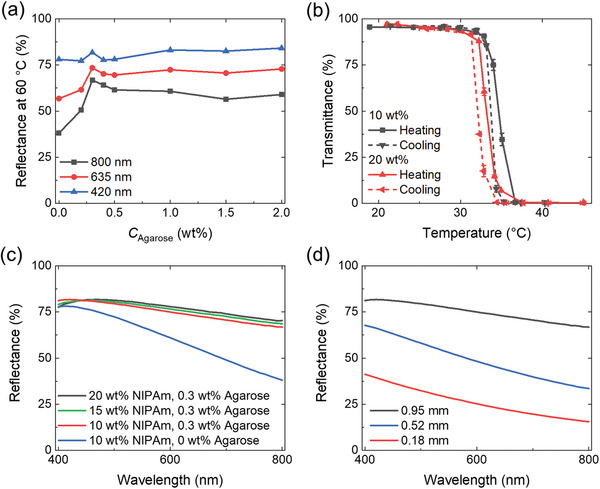
Influence of the hydrogel composition. a) The effect of agarose concentration (*C*_agarose_) on the total reflectance at 60 °C of the hydrogel films with as‐prepared thickness of 1 mm. Lines are to guide the eye. b) The effect of monomer concentration on the phase transition of the hydrogel upon heating (solid lines) and cooling (dashed lines). c) The effect of NIPAm monomer concentration on the total reflectance of hydrogel films at 60 °C. d) The effect of the film thickness on the reflectance at 60 °C. The thickness refers to the shrunken thickness of the samples during the measurement. Unless otherwise specified, all samples were prepared using 10 wt% of NIPAm, 0.3 wt% of agarose, and 0.05 mol% of PEGDA with an as‐prepared thickness of 1 mm and a shrunken thickness between 0.86–0.95 mm (see Figure S3 in the Supporting Information).

The influence of the hydrogel composition on its optical properties and phase transition has been systematically studied and summarized in **Figure** **2**. In Figure 2a, the total reflectance of the ch‐PNIPAm hydrogel films (as‐prepared thickness: 1 mm) at 60 °C is presented at different wavelengths as a function of agarose concentration. It is observed that for all wavelengths the reflectance increases significantly as the concentration of agarose increases to 0.3 wt%. Interestingly, this is also the lowest concentration to achieve gelation of the agarose used in this study,^[^ ^34^ ^]^ indicating the importance of a percolated physical network for the enhanced whiteness of the hydrogel. The reflectance reaches a local maximum at an agarose concentration of 0.3 wt%, above which slightly lower values can be observed for 800 and 635 nm (Figure 2a). At 420 nm, the maximum reflectance of 84% is achieved on the sample with 2 wt% agarose, compared to 82% from the sample with 0.3 wt% agarose. While the fibril diameter of agarose is almost independent on its concentration,^[^ ^33^ ^]^ the network density (concentration) seems to play a minor role in determining the reflectance of the channeled hydrogel.

Figure 2b,c presents the effect of the monomer concentration on the LCST and reflectance of the hydrogel films at 60 °C, respectively. When the monomer concentration is increased from 10 to 20 wt%, the LCST decreases by 1.5 °C from 34.7 to 33.2 °C. For both monomer concentrations there is a small hysteresis of roughly 1 °C between the LCSTs measured for heating and cooling, as commonly observed in the phase transitions of hydrogels.^[^ ^38^ ^]^ On the other hand, the NIPAm monomer concentration does not significantly affect the reflectance at 60 °C, which slightly increases by 2.5% above 500 nm as the monomer concentration increases from 10 to 20 wt%. In contrast, the reflectance changes dramatically with the film thickness (Figure 2d), a phenomenon that is well known for light‐scattering media.^[^ ^5^ ^]^ For the hydrogel film with a shrunken thickness of 0.18 mm, the reflectance is 41% at 400 nm and 15% at 800 nm, compared to 81% and 67% for 0.95‐mm‐thick film, respectively.

To determine whether the enhancement of the whiteness is universal for different PNIPAm‐based interpenetrating network hydrogels and whether the removal of the first network is relevant, different interpenetrating network gels were made. First, samples were prepared using polyacrylamide hydrogel or cellulose nanofibril hydrogel produced using (2,2,6,6‐Tetramethylpiperidin‐1‐yl)oxyl (TEMPO) mediated oxidation (TEMPO‐NFC), replacing agarose as the first network. However, these samples did not show significantly enhanced whiteness compared to the channeled hydrogel using agarose (Figure S6, Supporting Information). On the other hand, agarose can be used to prepare channeled networks with other LCST hydrogels, such as P(DEGMA‐co‐OEGMA),^[^ ^39^ ^]^ or poly(*N*‐vinylcaprolactam)^[^ ^40^ ^]^ as the second crosslinked network, which all show enhanced whiteness above the LCST compared to pure hydrogels (Figure S7, Supporting Information). Together with the fact that the reflectance of the hydrogel is much lower if the agarose is not gelled before the polymerization of the PNIPAm network (Figure S8, Supporting Information), it can be proposed that the percolated agarose network serves as a physical template and modifies the microscopic structure of the second hydrogel network by introducing nanoscopic channels, which lead to the enhanced whiteness of the PNIPAm network above the LCST without affecting much the transparency at room temperature.

### 2.3 Micromorphology of the ch‐PNIPAm Hydrogel

**Figure 3:**
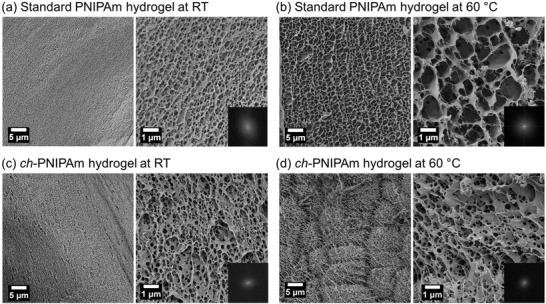
Microstructure characterization of the hydrogel using scanning electron microscopy (SEM). Standard PNIPAm at a) room temperature (RT) and at b) 60 °C. ch‐PNIPAm hydrogel at c) room temperature and at d) 60 °C. All gels prepared using 10 wt% NIPAm and 0.05 mol% PEGDA. ch‐PNIPAm hydrogel prepared using 0.3 wt% agarose. Inset: Fast Fourier Transform (FFT) image of the corresponding SEM image.

To characterize the micromorphology of the hydrogel, the samples equilibrated at room temperature and 60 °C were shock‐frozen using liquid propane and then lyophilized to preserve the micromorphology of the network. Liquid propane was used instead of liquid nitrogen to provide a faster cooling rate and minimize the formation of ice crystals, allowed by the reduced Leidenfrost effect.^[^ ^41^ ^]^ SEM measurements were then carried out on the lyophilized ch‐PNIPAm hydrogel and, as a reference, also on the standard PNIPAm hydrogel. The results are summarized in **Figure** **3**. At room temperature, the ch‐PNIPAm (Figure 3c) and standard PNIPAm (Figure 3a) hydrogels exhibit similar structural features with polydisperse pores, though the ch‐PNIPAm hydrogel shows slightly larger periodicity as seen from the FFT images of the corresponding SEM images (insets of Figure 3). At 60 °C, both the ch‐PNIPAm and standard PNIPAm hydrogel underwent a significant change in the micromorphology, showing an increase in the average pore size. However, the difference between the two hydrogels becomes more pronounced: the pores of the standard PNIPAm hydrogel (Figure 3b) show a broad, ill‐defined distribution up to several micrometers in diameter (Figure S9, Supporting Information). In contrast, the pores of the ch‐PNIPAm hydrogel (Figure 3d) show a more defined distribution in the sub‐micron scale, which is advantageous for light scattering at the visible wavelengths.^[^ ^4^, ^5^ ^]^ This difference in the porosity is the key for the enhanced whiteness due to two factors: the interfacial scattering area is much higher in the ch‐PNIPAm hydrogel, and the sub‐micron pores are more efficient in scattering visible light.^[^ ^4^, ^5^ ^]^ Based on the observation above, it can be postulated that the bright whiteness in the ch‐PNIPAm is a result of the channel formation by agarose, which facilitates water transport within the hydrogel and thus faster phase transitions.^[^ ^42^ ^]^ As a result, smaller pores are formed inside the ch‐PNIPAm above the LCST that give rise to enhanced scattering of visible light. Though the lyophilized hydrogel remains transparent after re‐hydration, indicating the absence of significant structural changes in the hydrogel, it should be noted that artifacts in the microscopic structures of the hydrogel, such as the collapse of polymer network, may be produced during the shock‐freezing and lyophilization process. Therefore, the above‐discussed results are mainly aimed at providing a qualitative explanation for the enhanced whiteness in the ch‐PNIPAm. Further quantitative and noninvasive characterization of the porosity of the hydrogel can be done using, e.g., small angle X‐ray scattering or laser speckle measurements,^[^ ^9^ ^]^ which will be investigated in our future studies.

### 2.4 Phase Transition Kinetics via Photothermal Switching

**Figure 4:**
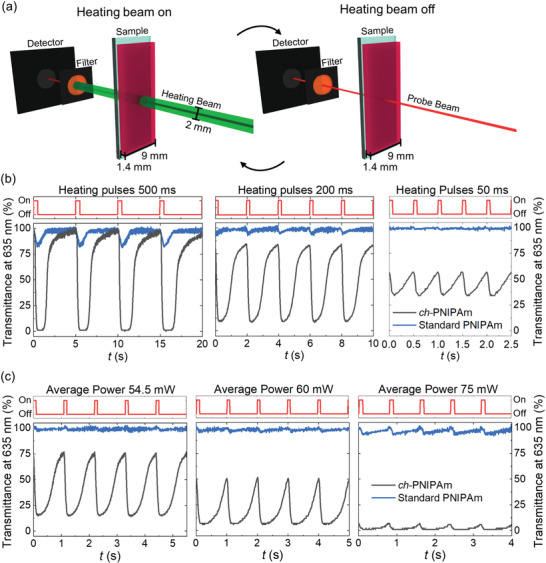
Kinetics of the hydrogel phase transition upon photothermal heating. a) Illustration of the setup used for photothermal heating and in situ measurement of the transmittance. The hydrogel film was attached on a transparent glass substrate and placed in a cuvette (not shown) filled with water. The hydrogel appears red due to the presence of AuNPs. b) The measured transmittance changes of ch‐PNIPAm and standard PNIPAm hydrogels upon different heating pulses. The laser power *P*_0_ = 600 mW (19.1 W cm^−2^), *P*_avg_ = 60 mW (1.9 W cm^−2^). c) Transmittance change of the ch‐PNIPAm and standard PNIPAm hydrogels at different average powers. *P*_0_ = 600 mW (19.1 W cm^−2^), *t*_on_ = 100 ms.

As a result of the nanoscopic channels formed inside the hydrogel,^[^ ^42^ ^]^ a notable feature of the ch‐PNIPAm hydrogel is its fast phase transition kinetics as shown in **Figure** **4**. Conventionally, the kinetics of a hydrogel is measured by the volume or transmittance change upon temperature jumps in water baths.^[^ ^38^, ^43^, ^44^, ^45^, ^46^ ^]^ The generally slow response speed is due to two factors that affect the kinetics: slow temperature change of the sample and skin layer formation that slows down the water diffusion. To achieve fast switching, laser‐induced photothermal heating has been implemented to achieve local temperature jumps within the excitation light spot and thus the modulation of the transmission or volume of the gel.^[^ ^16^, ^47^, ^48^, ^49^, ^50^ ^]^ Herein, PEGylated gold nanoparticles (AuNPs) with an average diameter of 19 nm were synthesized and incorporated into the hydrogels as efficient photothermal converters. TEM images and UV–vis spectra of the AuNPs are presented in Figure S10 in the Supporting Information, and details on the synthesis and modification of the AuNPs can be found in Experimental Section. The ch‐PNIPAm hydrogel films containing AuNPs were irradiated with modulated laser at 532 nm, near the absorption maximum of the AuNPs (Figure S11, Supporting Information), and the response of the hydrogel (transmittance change) on the irradiated spot was recorded using a probe laser beam at 635 nm. The transmitted heating beam at 532 nm was filtered out before the photodetector to ensure that only the probe beam was measured. A scheme of the setup is presented in Figure 4a. The parameters used to study the material kinetics were laser power (*P*_0_), laser pulse length (*t*_on_), interval between laser pulses (*t*_off_), and the average laser power (*P*_avg_), defined as

(1) $$P_{avg}=P_{0}\;\cdot \;\frac{t_{on}}{t_{on}+t_{off}}$$

In Figure 4b,c, the photothermally induced changes in the transmittance of both ch‐PNIPAm and standard PNIPAm films are presented. It should be noted that 100% transmittance is defined as the transmittance through the sample at room temperature. At the laser power of 600 mW and the *t*_on_:*t*_off_ ratio of 1:9 (*P*_avg_ = 60 mW), the effect of the duration of the heating pulse is demonstrated in Figure 4b. It can be seen that longer pulses result in larger transmittance changes. Within 500 ms of irradiation, the *ch*‐PNIPAm hydrogel can undergo an almost 100% change in the transmittance from a highly transparent to a nearly opaque state, while 4.5 s is needed for the hydrogel to recover the transparency. The relatively long recovery time compared to the excitation time could have resulted from a slow temperature equilibration with the surrounding water during cooling, as has been observed also in other photothermally controlled hydrogel‐based systems.^[^ ^16^ ^]^ During the short period of strong light irradiation, the hydrogel temperature is quickly increased within a small volume centered at the laser spot, while not reaching its thermal equilibrium state. During cooling, however, it requires the thermal equilibration of the heated volume with the surrounding environment, resulting in a comparatively slow relaxation process. It is important to note that, for irradiation as short as 50 ms, the ch‐PNIPAm hydrogel can still respond with a 23% change in the transmittance. In contrast, standard PNIPAm gel did not react to the same excitation in terms of amplitude and speed. For 500 ms excitation pulse, the standard PNIPAm hydrogel only showed a 16% change in the transmittance, while below 200 ms, the transmittance remained practically unchanged. The ch‐PNIPAm hydrogel demonstrates that its macroscopic optical properties can be modulated at a time scale down to tens of milliseconds using local photothermal heating, compared to minutes in conventional temperature‐jump experiments. This enhanced kinetics of the ch‐PNIPAm hydrogel can be attributed to the channels formed by using agarose as the template, which improve water transport inside the hydrogel and thus accelerates the phase separation process during phase transition.^[^ ^42^, ^51^, ^52^, ^53^, ^54^, ^55^, ^56^, ^57^, ^58^, ^59^ ^]^ Control experiments show that the ch‐PNIPAm also has a relatively fast volume change upon phase transition, but the kinetics is slower than that of the transparency change (Figure S12, Supporting Information). These results indicate that the porous channels may have served as a temporary reservoir for water during the initial stage of phase transition, which leads to fast microscopic phase separation and thus the change in whiteness. At a longer time scale, the water is expelled from the hydrogel through the channels, leading to the shrinking of the hydrogel.

Figure 4c shows the influence of the average power on the transmittance change of the hydrogels at fixed *P*_0_ (600 mW) and *t*_on_ (100 ms). In principle, higher average power leads to a higher average temperature, around which the temperature oscillates according to the modulation. This can be observed in Figure 4c, where the oscillation of transmittance gradually approaches 0% as the average power increases. At an average power of 75 mW, the transmittance stays almost unchanged near 0%, indicating that the temperature of the system is mostly above the LCST upon modulation. At an average power of 54.5 mW, the amplitude of the transmittance oscillation is the highest, reaching a 62% change in the transmittance, indicating that the temperature is oscillating around the LCST. From Figure 4b,c, it can be concluded that the ch‐PNIPAm hydrogel has much faster phase transition kinetics than standard PNIPAm hydrogels and the modulation of the transmittance can be controlled by varying the laser power (*P*_0_), pulse duration (*t*_on_), and average power (*P*_avg_). For a comprehensive summary of the influence of these parameters, see Figures S13–S19 in the Supporting Information.

To quantify the kinetics of the whiteness change in PNIPAm hydrogels, single‐exponential fitting was performed on the acquired kinetic measurement data,^[^ ^47^, ^60^, ^61^ ^]^ to determine the time constant (τ). For the ch‐PNIPAm hydrogel, the resulting τ for the transparency decrease was determined to be 87 ± 11 ms (7 sets of data), while for the standard PNIPAm hydrogel it was 1580 ± 160 ms (12 sets of data). Thus, the transition kinetics of the ch‐PNIPAm hydrogel is 18 times faster than that of standard PNIPAm. For the details of the fitting, see the text and Figure S20 in the Supporting Information. This switching speed is comparable to or even faster than existing electrochromic devices, which typically show a response time in the second to sub‐second range.^[^ ^35^, ^62^, ^63^ ^]^ In comparison, the τ was 2 min for a photothermally active PNIPAm/graphene oxide composite (5 mm × 25 mm disk),^[^ ^64^ ^]^ 8.7 min for externally heated PNIPAm/silk fibroin hydrogel (3 mm × 12 mm disk),^[^ ^26^ ^]^ and roughly 1 s for photothermally controlled PNIPAm fiber mats (8 mm × 15 mm × 0.3 mm).^[^ ^65^ ^]^

### 2.5 Switchable Bright Whiteness for Color Display

**Figure 5:**
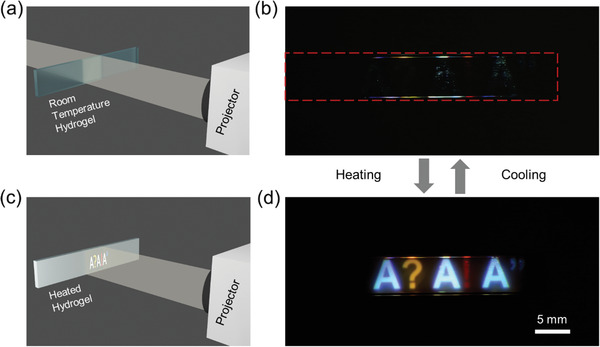
A switchable projection screen based on the high whiteness of ch‐PNIPAm hydrogel. a) Illustration of the capillary containing the ch‐PNIPAm hydrogel and image projection on the transparent gel at room temperature. b) Photograph of the hydrogel at room temperature with the image being projected on the hydrogel. Red‐dashed frame shows the boundary of the glass capillary containing the hydrogel. The scattered light is due to the dust particles and edges of the capillary. c) Illustration of the projected image on the hydrogel above LCST. d) Photo of the displayed image on the hydrogel. Thickness of the ch‐PNIPAm hydrogel: 500 µm.

Previously, materials that are capable of changing their transparency have been utilized in smart/adaptive windows applications.^[^ ^8^, ^20^, ^21^, ^66^ ^]^ Due to the switchable whiteness and fast kinetics in phase transition, the ch‐PNIPAm hydrogel is also highly desirable for optical applications. Herein, we demonstrate a color display using switchable whiteness in ch‐PNIPAm hydrogel, where an image projected onto the transparent hydrogel window only shows up when the temperature is above the LCST. At room temperature (**Figure** **5**a), the hydrogel is highly transparent, and the projected light passes through the hydrogel without being reflected or scattered, therefore leaving the image invisible (Figure 5b). Once heated above the LCST (Figure 5c,d), the projected image is clearly displayed on the hydrogel film with both its shape and color well preserved due to the efficient and uniform scattering of the hydrogel. Video S1 in the Supporting Information shows the reversible switching process of the hydrogel screen using a heat gun. In this case, the switching time is limited by the heat equilibration in the system. Therefore, the switching time can be significantly accelerated by employing local heating method such as joule heating using electric current, for instance with indium tin oxide‐coated glass substrates. The encapsulation of the hydrogel with a glass capillary shown here demonstrates the applicability of the system in a dry environment, in addition to the intrinsic suitability in underwater applications. Furthermore, due to its softness and stretchability, the ch‐PNIPAm hydrogel may also be used for flexible optical devices.

## 3 Conclusions

Using agarose as the first physical template in a semi‐interpenetrating network, we show that hydrogels with percolated porous channels can be prepared, which display bright whiteness above the LCST. For the ch‐PNIPAm, the reflectance above the LSCT can reach 75–82% between 400 and 600 nm for the film thickness of 0.95 mm with a polymer content of around 10 wt%, which is comparable to the whiteness found in biological aqueous systems such as the cuttlefish *Sepia officinalis*. Besides, the ch‐PNIPAm hydrogel shows a sharper phase transition and 18 times faster kinetics compared to standard PNIPAm hydrogel, as characterized by the transmittance change upon equilibrium or photothermal heating. The ch‐PNIPAm hydrogel is capable of responding to irradiation pulses as short as 50 ms with a change of 23% in the transmittance, while standard PNIPAm shows no response for pulse duration shorter than 100 ms under the same conditions. The strategy of using agarose to prepare channeled hydrogels is also applicable to other LCST hydrogel systems besides the PNIPAm, such as poly(*N*‐vinylcaprolactam), where the reflectance of the hydrogel can be significantly enhanced in the visible range to give a whiter appearance. The results demonstrate that bright whiteness and fast kinetics can be achieved in hydrogels as a result of the nanoscopic channels formed using agarose as the template inside the hydrogel, which facilitates water transport and the formation of smaller pores inside the hydrogel upon phase transition. The demonstrated bright whiteness of the hydrogel sheds light on an aspect that is often neglected due to the low refractive index contrast and ill‐defined porosities in the hydrogel. This opens up new application opportunities such as the demonstrated switchable color display, where an image projected onto the hydrogel is only visible when the temperature is above the LCST of the hydrogel. As the polymer content in the hydrogel is less than 10 wt%, there is also plenty of room for further engineering of the optical properties of the hydrogel by incorporating, for instance, plasmonic nanoparticles, or photochromic groups to achieve advanced optical functionalities.

## 4 Experimental Section

##### Materials

*N*‐isopropylacrylamide (99%) was purchased from Fisher Scientific and recrystallized from *n*‐hexane (≥ 97%, Sigma‐Aldrich). Poly(ethylene glycol) diacrylate (PEGDA, *M_n_* = 10 000), *N*,*N′*‐Methylenebisacrylamide (≥ 99.5%), *N*‐vinylcaprolactam (98%), acrylamide (≥ 99%), di(ethylene glycol) methyl ether methacrylate (95%), poly(ethylene glycol) methyl ether methacrylate (*M*_w_ = 500), agarose (Ultra‐low Gelling Temperature, A5030), high melting point agarose (A2576), isopropanol (≥ 99.5%), gold(III) chloride trihydrate (HAuCl4·3H_2_O, > 99.9% trace metals basis), sodium citrate tribasic dihydrate (BioUltra, 99.5%), poly(ethylene glycol) methyl ether thiol (PEG‐SH, *M_n_* = 2000), 3‐(trimethoxysilyl)propyl acrylate (≥ 92%), 2,2,6,6‐Tetramethylpiperidin‐1‐yl)oxyl (TEMPO, 98%), and 2‐hydroxy‐4′‐(2‐hydroxyethoxy)‐2‐methylpropiophenone (photoinitiator, Irgacure 2959, 98%) were purchased from Sigma‐Aldrich. Sodium hypochlorite (12% Cl_2_ in aqueous solution) was purchased from VWR. Sodium bromide (99–100.5%), sodium hydroxide (97%) and hydrochloric acid (1 m) were purchased from Fisher Scientific. Ethanol (99.5%) was purchased from Altia Oyj, Finland. Deionized water (18.2 MΩ; DirectQ 3 UV; Millipore) was used in all experiments.

##### Functionalization of Glass Substrates

Glass slides (Corning, Sigma‐Aldrich) were cut into appropriate sizes and cleaned by sonication in isopropanol for 10 min. The surface of the slides was activated using oxygen plasma for 5 min (Pico, Diener Electronic GmbH, Germany) and functionalized by incubating the slides overnight in a desiccator containing 3‐(Trimethoxysilyl)propyl methacrylate (100 µL) in a petri dish at 8·10^−2^ mbar. On the next day the petri dish was removed, and the desiccator was pumped to 1·10^−3^ mbar for 2 h to remove the excess of silane. The silanized glass slides were stored in a sealed petri dish in the fridge and used within a week after preparation.

##### Synthesis and Modification of Gold Nanoparticles (19 nm)

Citrate‐stabilized gold nanoparticles (AuNPs) were prepared by the classical citrate reduction method.^[^ ^67^ ^]^ Trisodium citrate solution (2 mL, 1.0 wt%) was quickly injected to a boiling aqueous solution of HAuCl_4_·3H_2_O (100 mL, 0.01 wt%) under vigorous stirring. The solution was further refluxed for 10 min under stirring to complete the synthesis. The AuNPs were analyzed by transmission electron microscope (Tecnai 12), which showed an average diameter of 18.8 ± 2.0 nm, see Figure S10 in the Supporting Information for a representative image of the AuNPs. The 19 nm AuNPs were modified using PEG‐SH by adding an ethanolic PEG‐SH solution (2 mL, 5 mg mL^−1^) to the AuNP solution (50 mL), and the solution was shaken overnight on an orbital shaker. Subsequently, the modified AuNPs were purified by centrifugation (16 000 g for 40 min) and re‐dilution in pure water for three times. Finally, 1.5 mL of the concentrated PEGylated AuNPs was collected as a stock solution.

##### Preparation of TEMPO‐Oxidized Cellulose Nanofibrils (TEMPO‐NFC)

Bleached birch pulp (UPM Pietarsaari, Finland) was mixed with an aqueous solution of TEMPO (0.13 mmol g^−1^ pulp) and NaBr (4.65 mmol g^−1^ pulp) to a pulp dry matter content of 1.2 wt%. The pH of the solution was adjusted to 9.5 using 1 m NaOH, and a solution of NaClO (5 mmol g^−1^ pulp) was added stepwise while keeping the pH at 10.3 using 1 m HCl and 1 m NaOH. When the pH stabilized, the oxidation was stopped by addition of ethanol. The suspension was filtered, washed once with pure water, three times with water that had its pH adjusted to 2, and once more using pure water. The pulp cake was mixed with water to a concentration of 2 wt%, the pH was adjusted to 9 with 1 m NaOH and the pulp was passed through Microfluidizer M‐110P. The resulting clear gel was stored in a refrigerator.

##### Preparation of Hydrogel Films

To prepare the monomer solution, certain amount of agarose was dissolved in deionized water using a heat gun and vortexed until full dissolution. The agarose solution was then left to cool down at room temperature. In a typical procedure, agarose (3.38 mg) was dissolved in deionized water (900 µL), which was used to dissolve NIPAm (113 mg) and the photoinitiator (2.2 mg). The cross‐linker PEGDA (99.8 µL of 5 × 10^−3^ m aqueous solution) was added subsequently. The resulting solution contained 10 wt% NIPAm, 0.05 mol% of PEGDA relative to the NIPAm, and 0.3 wt% agarose. The solution was vortexed and then degassed by bubbling with nitrogen for 10 min. To prepare hydrogel films with a desirable thickness, a silanized glass slide was attached to a nonmodified slide using Parafilm (Parafilm M, Bemis) as the spacer. The film thickness was controlled by varying the number of Parafilm layers, for instance, 10 layers for 1 mm. The attached slides were placed into a glass vial, which was then purged with nitrogen for 5 min. The degassed monomer solution was then injected into the space between the attached glass slides, and the vial was further nitrogenated for 5 min and then placed in a fridge at 4 °C for 30 min for the gelation of agarose. Finally, the polymerization of NIPAm gel was carried out in a UV‐reactor (8 × 14 W lamps, 350 nm, Rayonet, USA) for 20 min. The resulting hydrogel films attached on the glass slides were purified by incubating the gels in a water bath at 60 °C for 30 min and re‐swelling in pure water at room temperature for 30 min. The process was repeated three times in total. In this way, the agarose and traces of unreacted reagents could be removed. Hydrogels with other compositions were prepared following the same protocol.

The hydrogel films containing AuNPs were prepared by replacing the water with the modified AuNP dispersion in the standard protocol to maintain the same final concentration of different components. The concentration of AuNP solution was adjusted by dilution with water, so that the optical density of the resulting films was 1 at 532 nm as determined by the UV–vis spectrometer.

The PAAm gel for PAAm‐PNIPAm interpenetrating network gel was prepared using 10 wt% of AAm and 1 mol% of BIS in the monomer solution. The resulting gel was swollen in degassed NIPAm‐BIS solution for two days before polymerisation to ensure uniform diffusion. The monomer solution was prepared to a NIPAm content of 10 wt% and 1 mol% BIS taking into account the water in the PAAm hydrogel. When using TEMPO‐NFC as the primary network, the monomer solution was prepared by using a solution of NIPAm and BIS to dilute TEMPO‐NFC so that the final concentrations of the constituents were 5 wt% of NIPAm, 1 mol% of BIS, and 0.5 wt% of TEMPO‐NFC. At this concentration, the TEMPO‐NFC formed a weak gel. Poly(di(ethylene glycol) methyl ether methacrylate‐co‐oligo(ethylene glycol) methyl ether methacrylate) (P(DEGMA‐co‐OEGMA)) was prepared using monomer molar ratio 1:5 (OEGMA:DEGMA) to a total monomer concentration of 15 wt%. PEGDA was used as the cross‐linker (0.1 mol%), and agarose content was kept at 1 wt% to enable gel handling. PVCL gel was prepared by dissolving agarose and cross‐linker in water, monomer in ethanol, and combining these solutions to produce 1:1 weight ratio of water and ethanol. The final monomer solution had 15 wt% VCL, 0.1 mol% PEGDA, and 1 wt% agarose. Under these conditions, agarose formed a slightly weaker gel than in pure water. Reference samples were prepared by replacing agarose solution with water. All of the gels were polymerized to a thickness of 5 mm in semimicro PMMA cuvettes (1.5 mL, BRAND, Sigma‐Aldrich) and washed following similar protocol to the film preparation.

##### UV–Vis–NIR Spectroscopy

UV–vis spectra between 400 and 800 nm were measured using a Cary 5000 (Agilent, USA) UV–vis spectrophotometer. The total reflectance of the films was measured in air using an integrating sphere (DRA 2500, Agilent) and the temperature of the sample was controlled using a TMS94 temperature controller (Linkam, UK). The transmittance of the films in water was measured as a function of temperature, which was controlled by a dual cell Peltier sample holder. The LCST was defined as the temperature at which the transmittance reached 50%. For the CIELAB color space, the total reflectance spectra were measured between 360 and 830 nm, and the calculation of the color space was conducted using the Scan program (version 6.2.0.1588) of the instrument.

##### FTIR Spectroscopy

The samples were prepared by drying the hydrogels in air for three days and then in vacuum (10^−3^ mbar) for five hours. The FTIR transmittance spectra were measured using Nicolet 380 (ThermoFisher Scientific, USA) with Smart Orbit Diamond ATR. The spectra were normalized for comparison.

##### SEM Characterization

Bulk gels were prepared in semimicro PMMA cuvettes using the same protocol as when preparing the films. The as‐prepared hydrogels had a dimension of 5 × 10 × 10 mm^3^. The bulk hydrogel was cut into small pieces (≈1 × 1 × 2 mm^3^) and equilibrated in water, either at room temperature or 60 °C water bath for 15 min. Subsequently, the gel pieces were shock‐frozen in liquid propane,^[^ ^41^ ^]^ fractured using a scalpel and lyophilized overnight (FreeZone −105 °C 4.5 Liter Cascade Benchtop Freeze Dry System, Labconco, USA). The lyophilized samples were attached to SEM stubs using carbon tape and sputter‐coated with 5 nm of Pt/Pd using EM ACE600 (Leica, Germany). The SEM Sigma VP (Zeiss, Germany) was used for the imaging of the fractured surfaces.

##### Analysis of Pore Size Distribution

The SEM images were first converted into binary ones using the Threshold function of ImageJ (V 1.52p). The binary images were then analyzed using the Analyze Particles function. Pores that were smaller than 200 nm (diameter) were excluded from the analysis, as they don't contribute much to the visible scattering. The whole process is shown in Figure S9 in the Supporting Information. The diameter of the pores was calculated from their area by assuming that they were perfectly round. More than 1000 pores were analyzed for the standard and the channeled PNIPAm at 60 °C.

##### Photothermal Modulation of Transmittance

Films containing AuNPs with an as‐prepared thickness of 0.5 mm were used for the photothermal heating experiments. After swelling, the film thicknesses were measured to be 1.36 mm for the ch‐PNIPAm gel and 1.45 mm for the standard PNIPAm gel. The films were placed in glass cuvettes (100‐OS, Hellma Analytics, Germany) filled with MilliQ water to prevent the gels from drying. The cuvette was placed between a mechanical shutter (SH1, Thorlabs, USA) and a photometer (PDA100A‐EC, Thorlabs, USA), which was connected to an oscilloscope (WaveSurfer 3074, Teledyne LeCroy, USA) to record the transmittance. A bandpass filter (632.8 ± 2 nm, FL632.8‐10, Thorlabs) was placed in front of the photometer in order to exclude the heating beam. Two laser beams were used: a 635 nm one (attenuated to 1 mW, MDL‐D‐635‐1W, Roithner Lasertechnik, Austria) as the probe beam and a 532 nm one (MGL‐F‐532‐2W, Roithner Lasertechnik, Austria) for the photothermal heating. Only the heating beam was controlled by the shutter, and the probe beam was always on. Due to the low absorbance of the AuNPs at 635 nm and the low laser power, the heating effect by the probe beam was negligible. The probe beam was aligned to the center of the heating beam in order to measure the transmittance change caused by the photoinduced phase transition of the hydrogel. For each measurement, a new spot on the sample was used to ensure consistency. The power of the heating laser was monitored in real time using a LabMax‐TO power meter (Coherent, USA) and the laser power variation was within 5% during the measurement. The 0% and 100% transmittance was calibrated for each measurement by recording the transmittance with and without blocking the probe beam. Therefore the 100% transmittance did not take into account the light absorbed or scattered by the gel and the cuvette.

##### Switchable Projection Screen

A film of the hydrogel containing 10 wt% NIPAm and 0.3 wt% agarose was synthesized inside a rectangular glass capillary with inner dimension of 0.5 × 5 × 50 mm^3^ (VitroTube 4905, Vitrocom). The hydrogel was prepared and washed in the same way as described above for the hydrogel films. A projector (PRO8530HDL, ViewSonic) was used to project the image on the hydrogel inside the capillary, which was held in front of a dark background. A heat gun was used to warm up the gel. Photos and videos were recorded with a digital camera (Canon EOS 5D Mark III, EF 100 mm f/2.8L).

## Conflict of Interest

The authors declare no conflict of interest.

## Supporting information

Supporting Information

Supplemental Video 1

## References

[1] P. Vukusic , B. Hallam , J. Noyes , Science 2007, 315, 348. 17234940 10.1126/science.1134666

[2] M. Burresi , L. Cortese , L. Pattelli , M. Kolle , P. Vukusic , D. S. Wiersma , U. Steiner , S. Vignolini , Sci. Rep. 2014, 4, 6075. 25123449 10.1038/srep06075 PMC4133710

[3] D. Xie , Z. Yang , X. Liu , S. Cui , H. Zhou , T. Fan , Soft Matter 2019, 15, 4294. 31095159 10.1039/c9sm00566h

[4] L. M. Mäthger , S. L. Senft , M. Gao , S. Karaveli , G. R. R. Bell , R. Zia , A. M. Kuzirian , P. B. Dennis , W. J. Crookes‐Goodson , R. R. Naik , G. W. Kattawar , R. T. Hanlon , Adv. Funct. Mater. 2013, 23, 3980.

[5] J. Syurik , R. H. Siddique , A. Dollmann , G. Gomard , M. Schneider , M. Worgull , G. Wiegand , H. Hölscher , Sci. Rep. 2017, 7, 46637. 28429805 10.1038/srep46637 PMC5399467

[6] B. Igic , L. D'Alba , M. D. Shawkey , Sci. Nat. 2018, 105, 18. 10.1007/s00114-018-1543-3 29445955

[7] X. Yao , Y. Hu , A. Grinthal , T. S. Wong , L. Mahadevan , J. Aizenberg , Nat. Mater. 2013, 12, 529. 23563739 10.1038/nmat3598

[8] L. Wang , H. K. Bisoyi , Z. Zheng , K. G. Gutierrez‐Cuevas , G. Singh , S. Kumar , T. J. Bunning , Q. Li , Mater. Today 2017, 20, 230.

[9] M. S. Toivonen , O. D. Onelli , G. Jacucci , V. Lovikka , O. J. Rojas , O. Ikkala , S. Vignolini , Adv. Mater. 2018, 30, 1704050. 10.1002/adma.201704050 29532967

[10] J. E. Stumpel , E. R. Gil , A. B. Spoelstra , C. W. M. Bastiaansen , D. J. Broer , A. P. H. J. Schenning , Adv. Funct. Mater. 2015, 25, 3314.

[11] A. J. J. Kragt , D. J. Broer , A. P. H. J. Schenning , Adv. Funct. Mater. 2018, 28, 1704756.

[12] V. Magdanz , G. Stoychev , L. Ionov , S. Sanchez , O. G. Schmidt , Angew. Chem., Int. Ed. 2014, 53, 2673. 10.1002/anie.201308610 PMC4255230 24481856

[13] Y. S. Zhang , A. Khademhosseini , Science 2017, 356, eaaf3627. 28473537 10.1126/science.aaf3627 PMC5841082

[14] Y. Chandorkar , A. Castro Nava , S. Schweizerhof , M. Van Dongen , T. Haraszti , J. Köhler , H. Zhang , R. Windoffer , A. Mourran , M. Möller , L. De Laporte , Nat. Commun. 2019, 10, 4027. 31492837 10.1038/s41467-019-11475-4 PMC6731269

[15] O. Erol , A. Pantula , W. Liu , D. H. Gracias , Adv. Mater. Technol. 2019, 4, 1900043.

[16] A. Mourran , H. Zhang , R. Vinokur , M. Möller , Adv. Mater. 2017, 29, 1604825. 10.1002/adma.201604825 27865006

[17] D. W. van Krevelen , K. te Nijenhuis , Properties of Polymers: Their Correlation with Chemical Structure, Their Numerical Estimation and Prediction from Additive Group Contributions, Elsevier, Amsterdam 2009.

[18] A. Halperin , M. Kröger , F. M. Winnik , Angew. Chem., Int. Ed. 2015, 54, 15342. 10.1002/anie.201506663 26612195

[19] Y. S. Yang , Y. Zhou , F. B. Yin Chiang , Y. Long , RSC Adv. 2016, 6, 61449.

[20] M. Wu , Y. Shi , R. Li , P. Wang , ACS Appl. Mater. Interfaces 2018, 10, 39819. 30365301 10.1021/acsami.8b15574

[21] T. G. La , X. Li , A. Kumar , Y. Fu , S. Yang , H. J. Chung , ACS Appl. Mater. Interfaces 2017, 9, 33100. 28836752 10.1021/acsami.7b08907

[22] M. Guvendiren , A. A. Soshinski , R. J. Gambogi , S. Yang , Polym. Eng. Sci. 2012, 52, 1317.

[23] W. J. Zheng , N. An , J. H. Yang , J. Zhou , Y. M. Chen , ACS Appl. Mater. Interfaces 2015, 7, 1758. 25561431 10.1021/am507339r

[24] J.‐Y. Sun , X. Zhao , W. R. K. Illeperuma , O. Chaudhuri , K. H. Oh , D. J. Mooney , J. J. Vlassak , Z. Suo , Nature 2012, 489, 133. 22955625 10.1038/nature11409 PMC3642868

[25] Z. Liang , C. Liu , L. Li , P. Xu , G. Luo , M. Ding , Q. Liang , Sci. Rep. 2016, 6, 33462. 27628933 10.1038/srep33462 PMC5024157

[26] E. S. Gil , S. M. Hudson , Biomacromolecules 2007, 8, 258. 17206815 10.1021/bm060543m

[27] E. S. Gil , S.‐H. Park , L. W. Tien , B. Trimmer , S. M. Hudson , D. L. Kaplan , Langmuir 2010, 26, 15614. 20804220 10.1021/la102509a

[28] C. Chang , K. Han , L. Zhang , Polym. Adv. Technol. 2011, 22, 1329.

[29] J. Wang , X. Zhou , H. Xiao , Carbohydr. Polym. 2013, 94, 749. 23544629 10.1016/j.carbpol.2013.01.036

[30] T. Kaneko , T. A. Asoh , M. Akashi , Macromol. Chem. Phys. 2005, 206, 566.

[31] K. Sakata , S. Taguchi , S. Uemura , M. Kunitake , S. Kawano , T. Nishimi , Chem. Lett. 2014, 43, 240.

[32] P. Aymard , D. R. Martin , K. Plucknett , T. J. Foster , A. H. Clark , I. T. Norton , Biopolymers 2001, 59, 131. 11391563 10.1002/1097-0282(200109)59:3<131::AID-BIP1013>3.0.CO;2-8

[33] K. Bertula , L. Martikainen , P. Munne , S. Hietala , J. Klefström , O. Ikkala , Nonappa , ACS Macro Lett. 2019, 8, 670. 10.1021/acsmacrolett.9b00258 35619522

[34] H. Zhang , H. Zeng , A. Priimagi , O. Ikkala , Nat. Commun. 2019, 10, 3267. 31332196 10.1038/s41467-019-11260-3 PMC6646376

[35] C. G. Wu , M. I. Lu , S. J. Chang , C. S. Wei , Adv. Funct. Mater. 2007, 17, 1063.

[36] S. Hirotsu , Y. Hirokawa , T. Tanaka , J. Chem. Phys. 1987, 87, 1392.

[37] Y. Li , T. Tanaka , Annu. Rev. Mater. Sci. 1992, 22, 243.

[38] M. Shibayama , T. Tanaka , in Advances in Polymer Science, Vol. 109, (Ed: Dušek K.), Springer, Berlin 1993, pp. 1–62.

[39] T. Kureha , K. Hayashi , M. Ohira , X. Li , M. Shibayama , Macromolecules 2018, 51, 8932.

[40] N. A. Cortez‐Lemus , A. Licea‐Claverie , Prog. Polym. Sci. 2016, 53, 1.

[41] D. Serp , M. Mueller , U. Von Stockar , I. W. Marison , Biotechnol. Bioeng. 2002, 79, 243. 12115412 10.1002/bit.10286

[42] Y. Kaneko , S. Nakamura , K. Sakai , T. Aoyagi , A. Kikuchi , Y. Sakurai , T. Okano , Macromolecules 1998, 31, 6099.

[43] A. Suzuki , S. Yoshikawa , G. Bai , J. Chem. Phys. 1999, 111, 360.

[44] Y. Li , T. Tanaka , J. Chem. Phys. 1990, 92, 1365.

[45] T. Okajima , I. Harada , K. Nishio , S. Hirotsu , J. Chem. Phys. 2002, 116, 9068.

[46] M. Shibayama , K. Nagai , Macromolecules 1999, 32, 7461.

[47] J. Zhao , H. Su , G. E. Vansuch , Z. Liu , K. Salaita , R. B. Dyer , ACS Nano 2019, 13, 515. 30574782 10.1021/acsnano.8b07150 PMC6467806

[48] S. Murphy , S. Jaber , C. Ritchie , M. Karg , P. Mulvaney , Langmuir 2016, 32, 12497. 27778508 10.1021/acs.langmuir.6b02781

[49] A. W. Hauser , A. A. Evans , J. H. Na , R. C. Hayward , Angew. Chem., Int. Ed. 2015, 54, 5434. 10.1002/anie.201412160 25752941

[50] Y. Zhou , A. W. Hauser , N. P. Bende , M. G. Kuzyk , R. C. Hayward , Adv. Funct. Mater. 2016, 26, 5447.

[51] J.‐T. Zhang , R. Bhat , K. D. Jandt , Acta Biomater. 2009, 5, 488. 18656431 10.1016/j.actbio.2008.06.012

[52] X. Z. Zhang , X. D. Xu , S. X. Cheng , R. X. Zhuo , Soft Matter 2008, 4, 385. 10.1039/b713803m 32907197

[53] Y. Zhao , X.‐J. Ju , L.‐P. Zhang , W. Wang , Y. Faraj , L.‐B. Zou , R. Xie , Z. Liu , L.‐Y. Chu , New J. Chem. 2019, 43, 9507.

[54] J.‐T. Zhang , S.‐X. Cheng , R.‐X. Zhuo , Colloid Polym. Sci. 2003, 281, 580.

[55] E. C. Cho , J.‐W. Kim , A. Fernández‐Nieves , D. A. Weitz , Nano Lett. 2008, 8, 168. 18085810 10.1021/nl072346e

[56] L. W. Xia , R. Xie , X. J. Ju , W. Wang , Q. Chen , L. Y. Chu , Nat. Commun. 2013, 4, 2226. 23900497 10.1038/ncomms3226 PMC3731657

[57] X.‐Z. Zhang , Y.‐Y. Yang , T.‐S. Chung , K.‐X. Ma , Langmuir 2001, 17, 6094.

[58] Q. Zhao , J. Sun , Q. Ling , Q. Zhou , Langmuir 2009, 25, 3249. 19437726 10.1021/la8038939

[59] J. Li , D. J. Mooney , Nat. Rev. Mater. 2016, 1, 16071. 29657852 10.1038/natrevmats.2016.71 PMC5898614

[60] J. Wang , D. Gan , L. A. Lyon , M. A. El‐Sayed , J. Am. Chem. Soc. 2001, 123, 11284. 11697971 10.1021/ja016610w

[61] E. Sato Matsuo , T. Tanaka , J. Chem. Phys. 1988, 89, 1695.

[62] C. Kortz , A. Hein , M. Ciobanu , L. Walder , E. Oesterschulze , Nat. Commun. 2019, 10, 4874. 31653835 10.1038/s41467-019-12617-4 PMC6814761

[63] X. W. Sun , J. X. Wang , Nano Lett. 2008, 8, 1884. 18564881 10.1021/nl0804856

[64] K. Shi , Z. Liu , Y. Y. Wei , W. Wang , X. J. Ju , R. Xie , L. Y. Chu , ACS Appl. Mater. Interfaces 2015, 7, 27289. 26580856 10.1021/acsami.5b08609

[65] C. L. Zhang , F. H. Cao , J. L. Wang , Z. L. Yu , J. Ge , Y. Lu , Z. H. Wang , S. H. Yu , ACS Appl. Mater. Interfaces 2017, 9, 24857. 28635250 10.1021/acsami.7b05223

[66] H. Zhu , L. Wang , Sol. Energy Mater. Sol. Cells 2019, 202, 110109.

[67] G. Frens , Nat. Phys. Sci. 1973, 241, 20.

