# Supplementary material for: Fast Switching of Bright Whiteness in Channeled Hydrogel Networks
Source: Adv Funct Mater. 2020 May 28;30(28):2000754. doi: 10.1002/adfm.202000754 (PMC7357574; doi:10.1002/adfm.202000754)
Supplement: Supplementary file 1 — Supporting Information [file ADFM-30-2000754-s001.pdf]

Copyright WILEY-VCH Verlag GmbH & Co. KGaA, 69469 Weinheim, Germany, 2020.

ADVANCED FUNCTIONAL MATERIALS

Supporting Information

for *Adv. Funct. Mater.,* DOI: 10.1002/adfm.202000754

Fast Switching of Bright Whiteness in Channeled Hydrogel Networks

Amanda Eklund, Hang Zhang, Hao Zeng, Arri Priimagi, and Olli Ikkala**

Copyright WILEY-VCH Verlag GmbH & Co. KGaA, 69469 Weinheim, Germany, 2020.

Supporting Information

Fast switching of bright whiteness in channeled hydrogel networks

Amanda Eklund, Hang Zhang, Hao Zeng, Arri Priimagi, and Olli Ikkala**

A. Eklund, Dr. H. Zhang, Prof. O. Ikkala
Department of Applied Physics, Aalto University, P.O. Box 15100, FI 02150 Espoo, Finland.
E-mail: hang.zhang@aalto.fi, olli.ikkala@aalto.fi

Dr. H. Zeng, Prof. A. Priimagi
Smart Photonic Materials, Faculty of Engineering and Natural Sciences, Tampere University, P.O. Box 541, FI-33101 Tampere, Finland.

Characteristic time of phase transition

The shrinking process can be described as a single-exponential process following equation:[61]

$$Trans(t) = Trans_{min} + \Delta Trans \times e^{-\frac{t-t_0}{\tau}}, \qquad \text{(S1)}$$

where $Trans(t)$ is the transmittance of the sample, $Trans_{min}$ the minimal value of the transmittance of the experiment, $\Delta Trans$ the overall change of transmittance (amplitude) of the experiment, t the time, t_0 the time at the beginning of the transmittance change, and τ the characteristic time of the transition. Among these parameters, $Trans_{min}$ and t_0 were determined manually and kept as constants, while $\Delta Trans$ and τ were determined by fitting the equation S1 to the data using OriginPro (Version 2019b, OriginLab Corporation, USA). An example of the fitting can be seen in **Figure S20**. The data with the change of transmittance over 90% was selected for the fitting in the case of the *ch*-PNIPAm hydrogel. The corresponding data for standard PNIPAm was chosen for fitting. The average τ for the *ch*-PNIPAm was 87 ms with the value varying between 75 and 102 ms when the fitting was performed to 7 sets of data. For standard PNIPAm the average τ was 1580 ms with the value varying between 1280 and 1830 ms among 12 sets of data. Regarding previously published results, the τ was calculated from the half-life $t_{1/2}$ of the shrinking process (time required for 50 % of the transmittance change to occur) using the equation below:[62]

$$\tau = \frac{t_{1/2}}{\ln 2}. \qquad \text{(S2)}$$

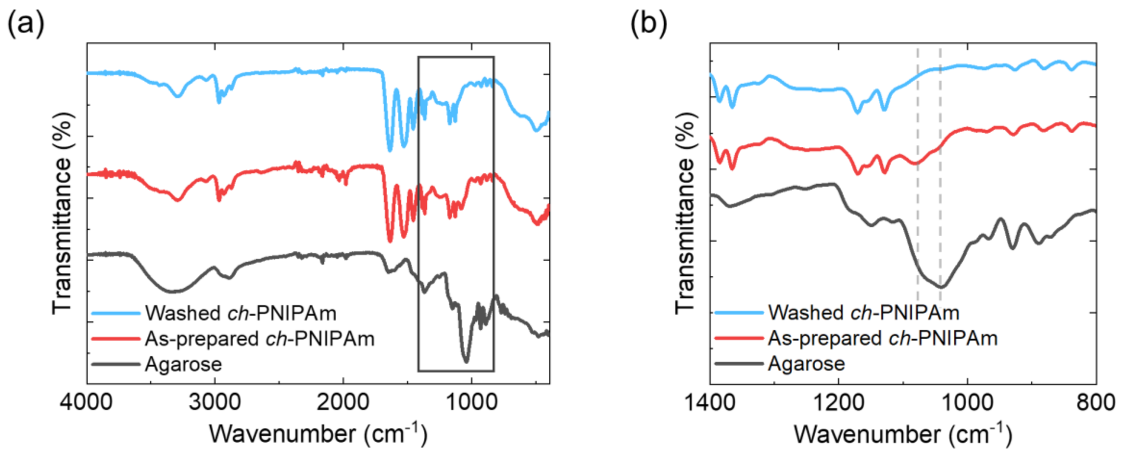

Figure S1. (a) The FTIR spectra of pure agarose, as-prepared *ch*-PNIPAm containing agarose, and washed *ch*-PNIPAm with agarose removed. (b) Magnification of the area 800-1400 cm^{-1} as marked by the frame in (a). The bands between 1000 and 1100 cm^{-1} (C-O stretching, marked by dashed lines) disappeared in the washed *ch*-PNIPAm, confirming the removal of the agarose. The *ch*-PNIPAm hydrogel was prepared using 10 wt% NIPAm, 0.05 mol% PEGDA (relative to NIPAm), and 0.3 wt% agarose.

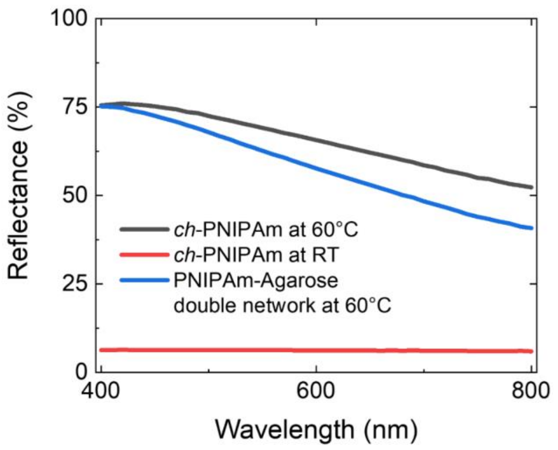

Figure S2. Effect of agarose removal on the reflectance of the hydrogel. PNIPAm-agarose double network hydrogel was prepared using 10 wt% NIPAm, 0.05 mol% PEGDA and 0.5 wt% high melting point agarose (T_m = 65.5 °C). The as-prepared thickness of the film was 1 mm. The double network hydrogel was washed at 80 °C to produce the *ch*-PNIPAm.

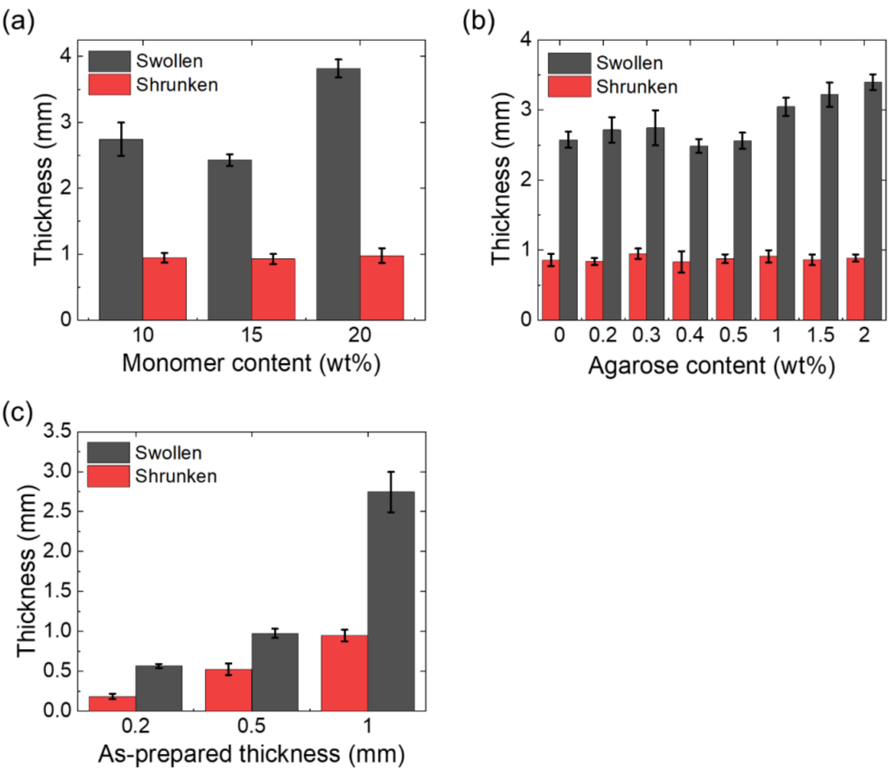

Figure S3. Effect of monomer concentration (a), agarose content (b), and the film preparation thickness (c) on the hydrogel film thickness in swollen and shrunken states. Unless otherwise specified, the monomer solutions contained 10 wt% NIPAm, 0.05 mol% PEGDA, and 0.3 wt% agarose, and the as-prepared thickness of the film was 1 mm.

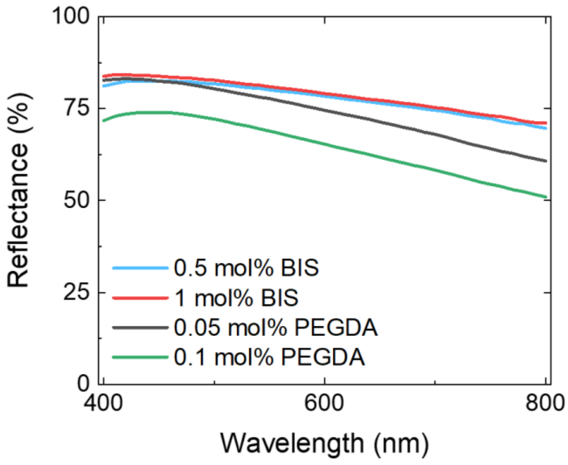

Figure S4. The effect of cross-linkers (BIS or PEGDA) on the reflectance of the *ch*-PNIPAm hydrogel films. All films were prepared using 10 wt% of NIPAm and 1 wt% of agarose in the monomer solution to a thickness of 1 mm.

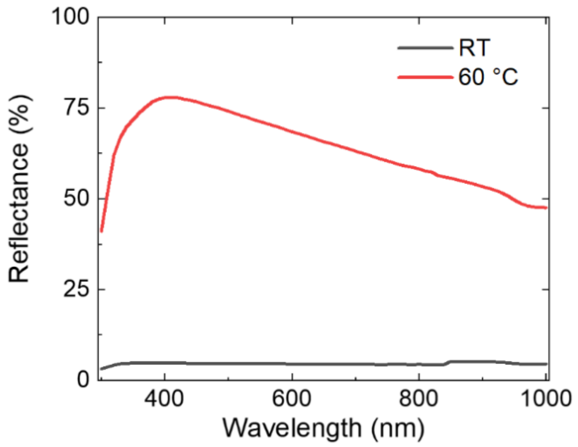

Figure S5. Total reflectance of the *ch*-PNIPAm hydrogel film between 300 nm and 1000 nm. The gel was prepared using 10 wt% of NIPAm, 0.05 mol% of PEGDA, and 0.3 wt% of agarose to a thickness of 1 mm.

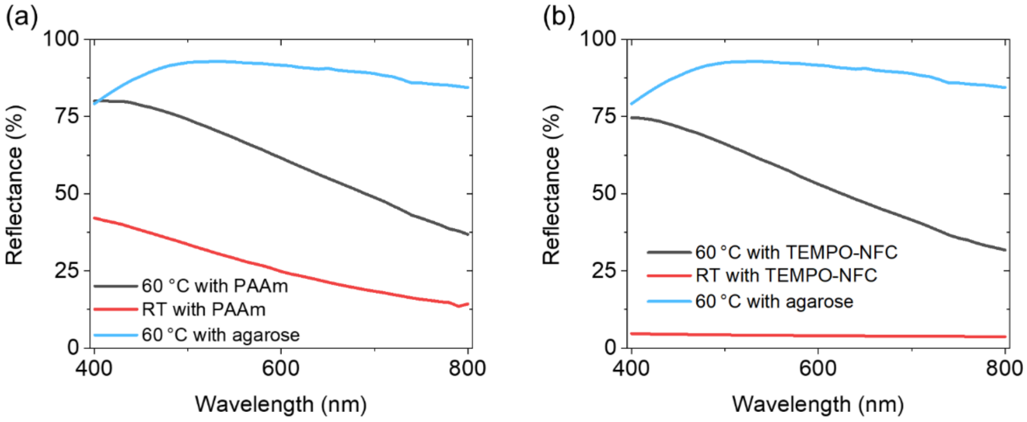

Figure S6. (a) Effect of chemically cross-linked PAAm primary network on the reflectance of bulk gel. (b) Effect of physically cross-linked TEMPO-NFC primary network on the reflectance of the bulk gel. The bulk hydrogel prepared with agarose as the primary network is shown as “60 °C with agarose” for comparison. All of the bulk gels had an as-prepared thickness of 5 mm.

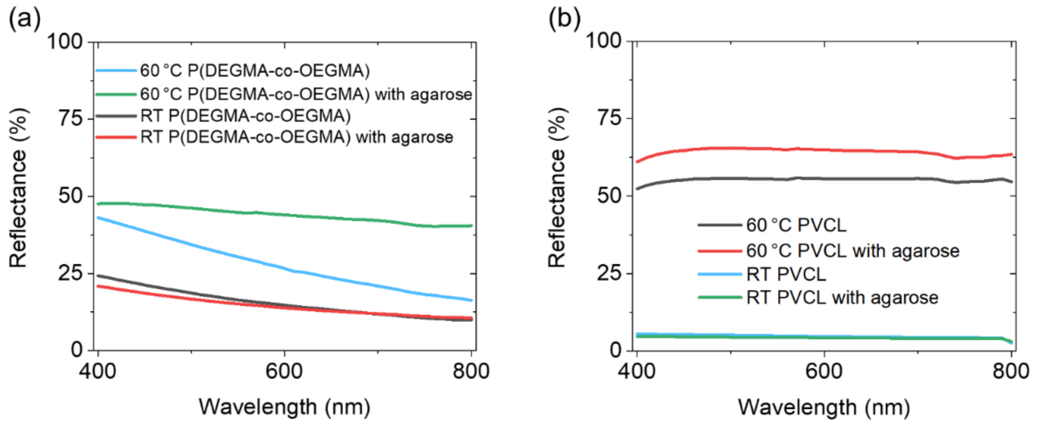

Figure S7. The effect of primary network of agarose on the reflectance of (a) P(DEGMA-co-OEGMA) bulk hydrogel and (b) PVCL bulk hydrogels. The gels prepared using agarose are compared to single network hydrogels of otherwise similar composition. All of the bulk gels had an as-prepared thickness of 5 mm.

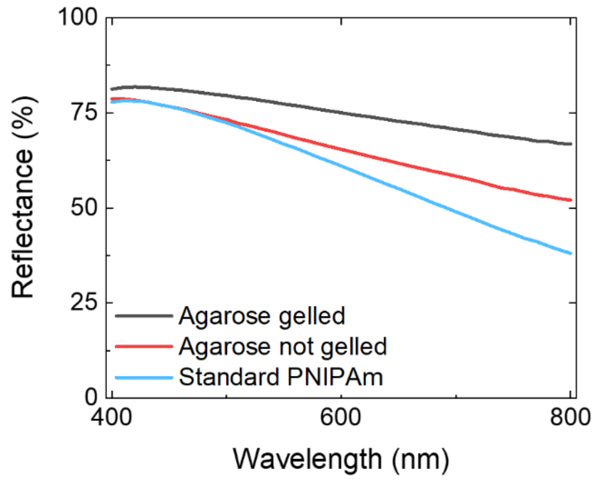

Figure S8. The effect of the agarose gelation on the film reflectance. All the films were prepared using 10 wt% of NIPAm and 0.05 mol% PEGDA. The films prepared using agarose had 0.3 wt% of agarose in the monomer solution. The sample produced without gelling the agarose was placed into the UV-chamber directly after preparation of monomer solution. All films had an as-prepared thickness of 1 mm.

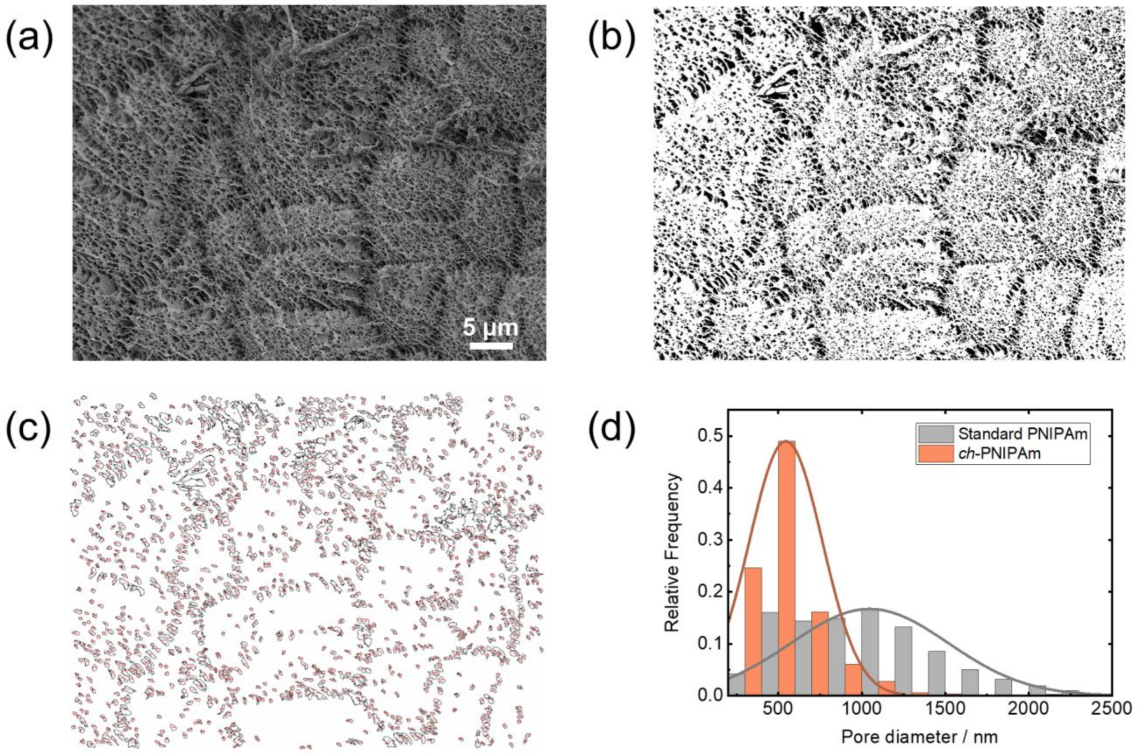

Figure S9. Analysis of the pore size distribution inside the hydrogel. (a) Original FESEM image of the *ch*-PNIPAm at 60 °C. (b) Binary image of (a) after thresholding. (c) Analyzed image of (b) showing the detected pores. Only the pores with diameter larger than 200 nm were measured. (d) Distribution of the pore diameter in standard and channeled PNIPAm hydrogel at 60 °C. More than 1000 pores were measured for each sample. The data were fitted with normal distribution.

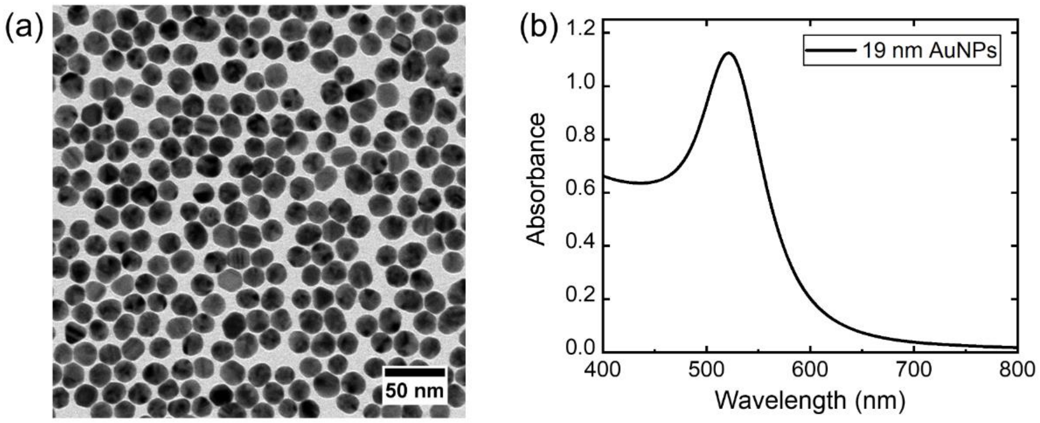

Figure S10. TEM image (a) and UV-Vis spectrum (b) of 19 nm gold nanoparticles (AuNPs).

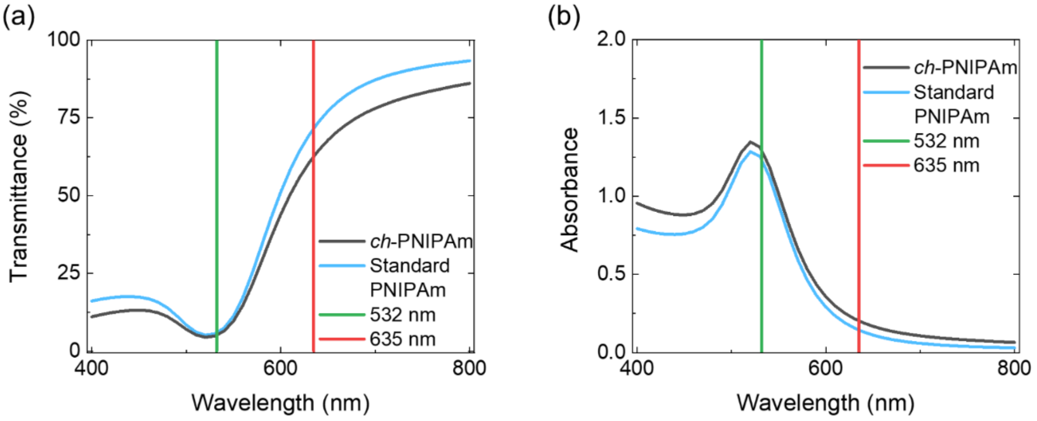

Figure S11. The transmittance (a) and absorbance (b) of the *ch*-PNIPAm and standard PNIPAm hydrogels prepared with gold nanoparticles in the monomer solution. The films were prepared using 10 wt% NIPAm, and 0.05 mol% of PEGDA to a thickness of 0.5 mm. The *ch*-PNIPAm was prepared using 0.3 wt% of agarose in the monomer solution.

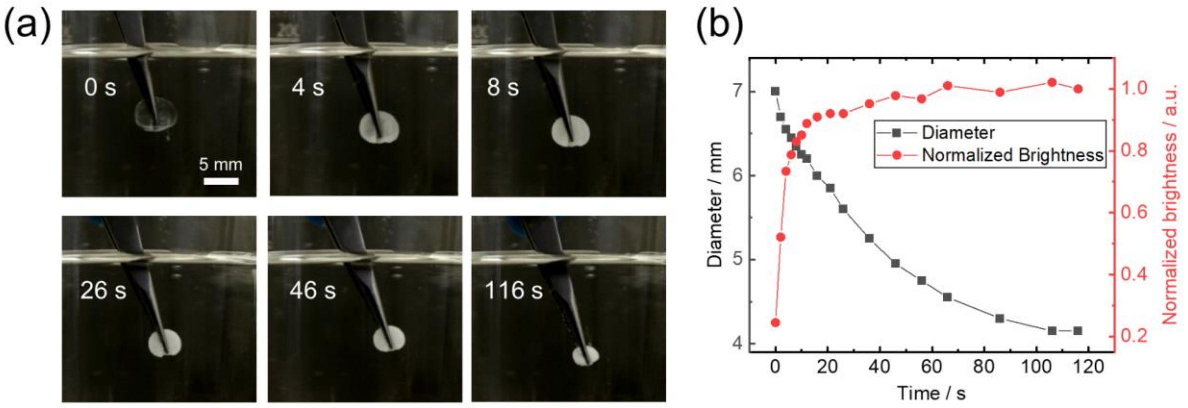

Figure S12. Phase transition of the *ch*-PNIPAm at 60 °C. (a) Image sequence showing a disk-shaped *ch*-PNIPAm in a water bath of 60 °C. (b) The kinetics of whiteness and volume change, represented by the normalized brightness of the hydrogel and the disk diameter, respectively. Both values were extracted from the video recording of the hydrogel. Sample size before immersion in water bath: 7 mm (diameter) × 3.3 mm (thickness).

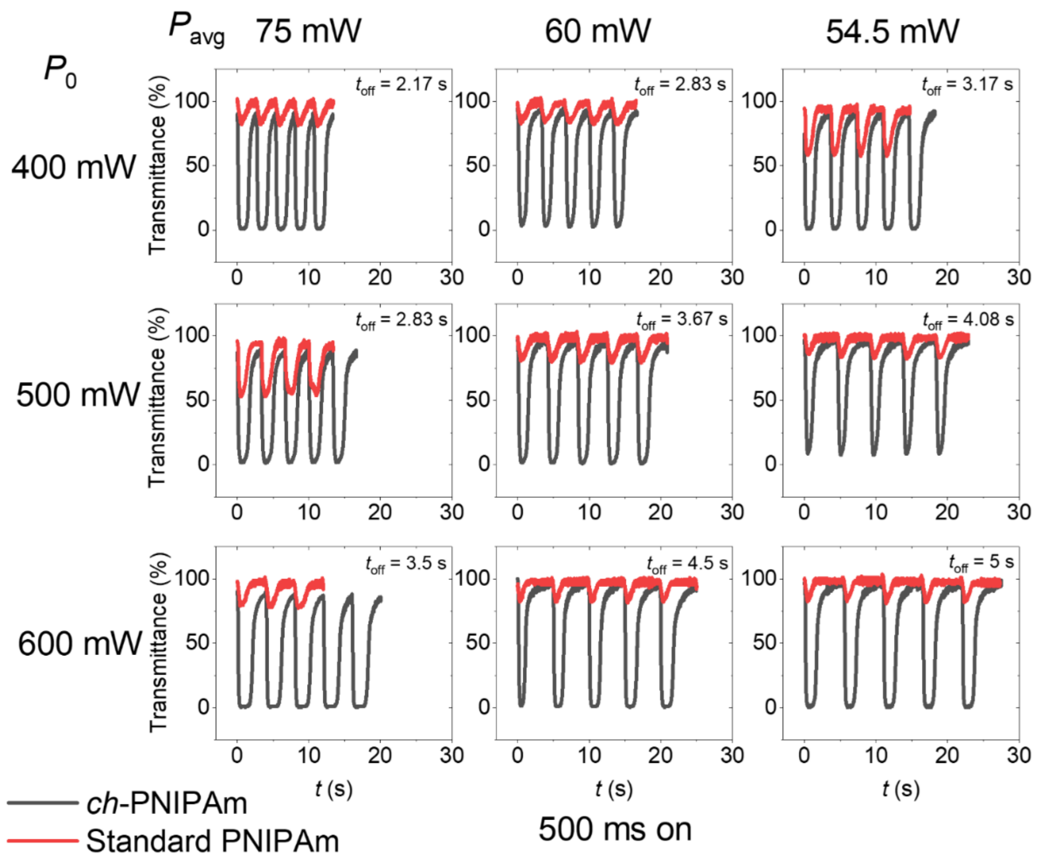

Figure S13. Comparison of the transmittance of *ch*-PNIPAm and standard PNIPAm hydrogel films at different laser powers (P_0) and average laser powers (P_{avg}) with the laser pulse length at 500 ms.

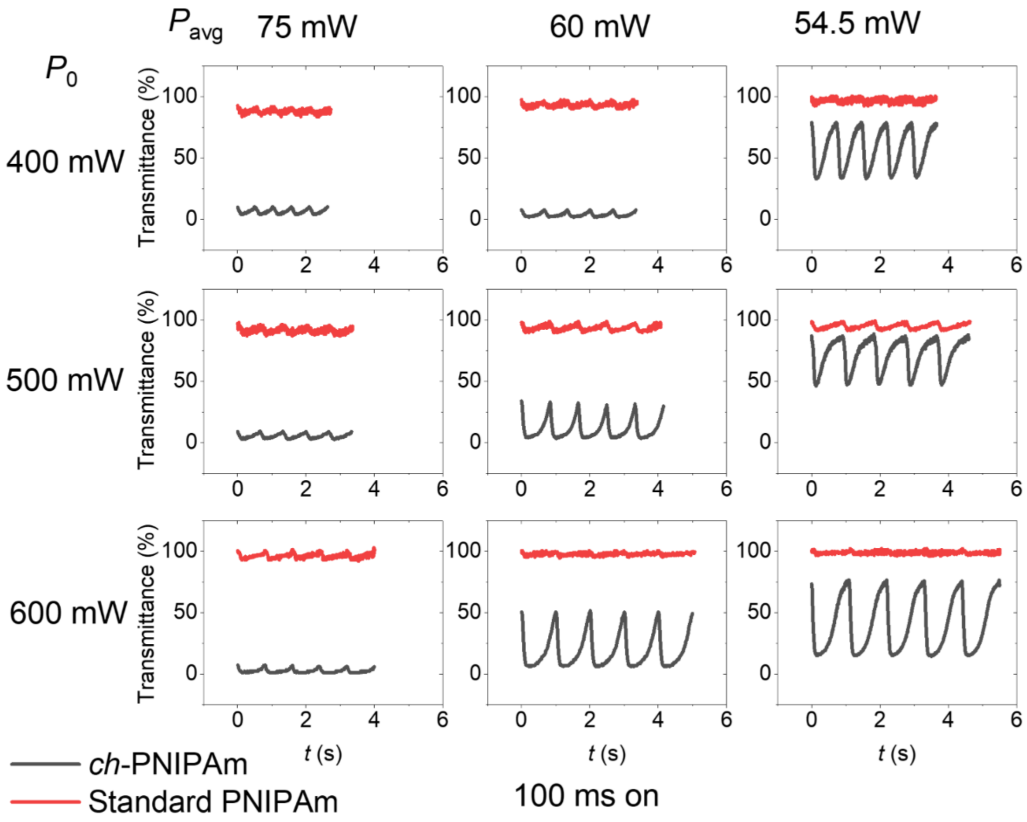

Figure S14. Comparison of the transmittance of *ch*-PNIPAm and standard PNIPAm hydrogel films at different laser powers (P_0) and average laser powers (P_{avg}) with the laser pulse length at 100 ms.

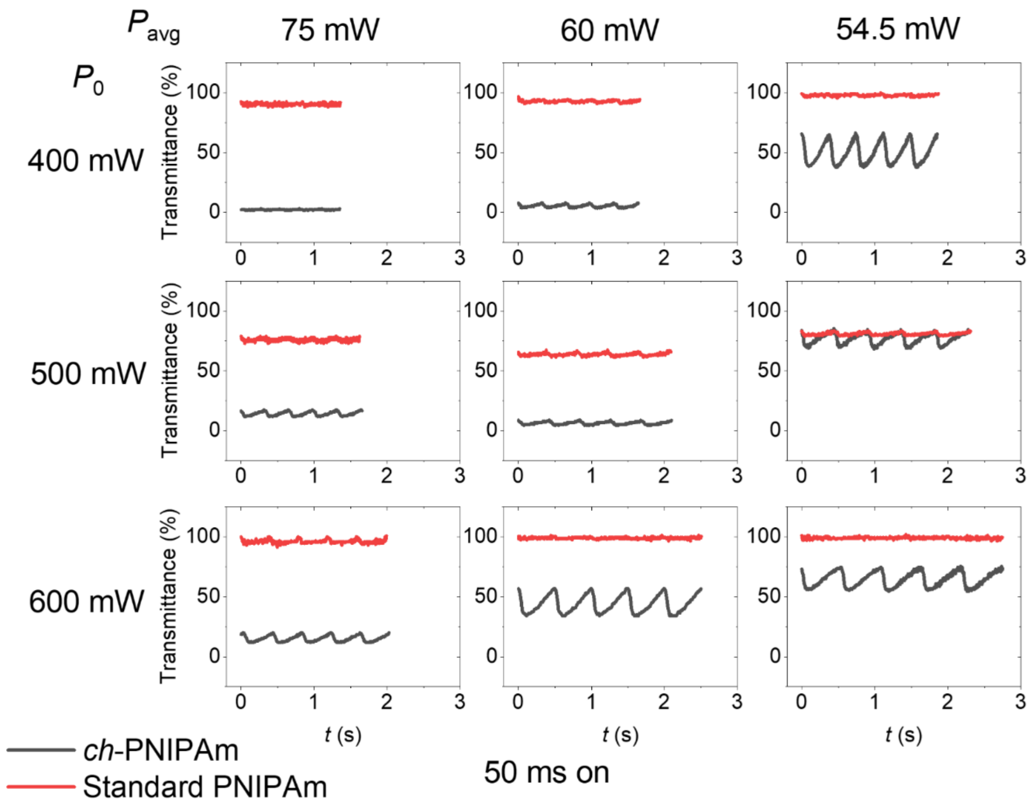

Figure S15. Comparison of the transmittance of *ch*-PNIPAm and standard PNIPAm hydrogel films at different laser powers (P_0) and average laser powers (P_{avg}) with the laser pulse length at 50 ms.

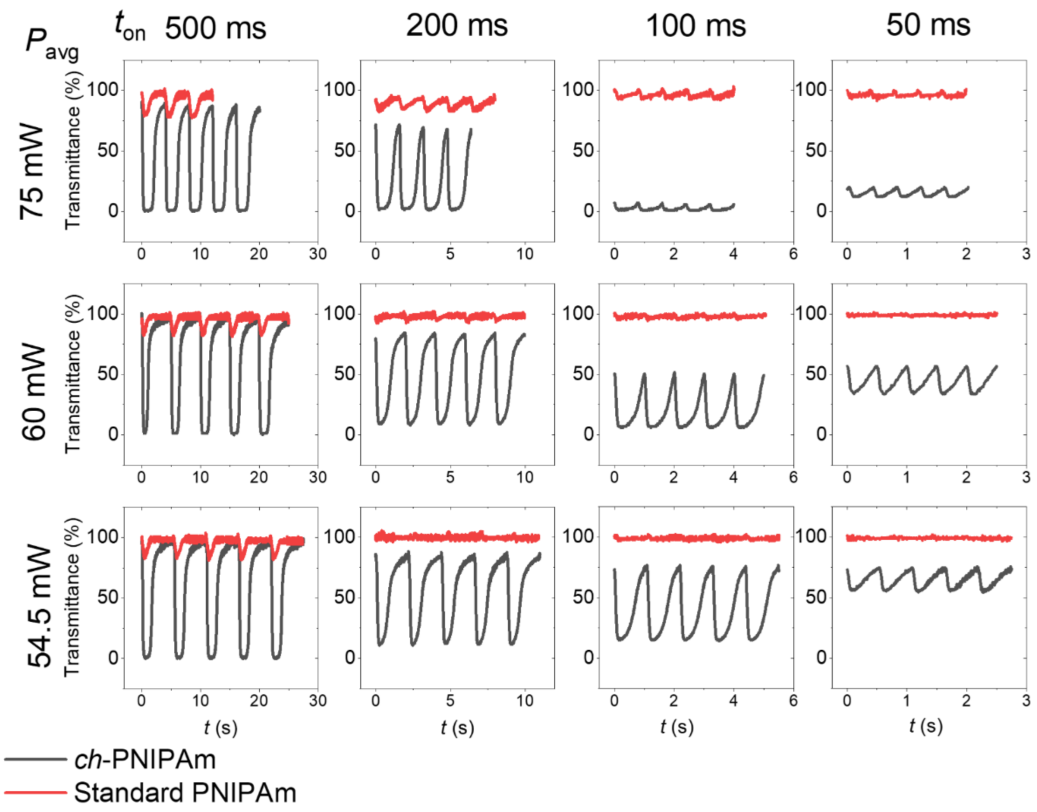

Figure S16. Comparison of the transmittance of *ch*-PNIPAm and standard PNIPAm hydrogel films at different laser pulse lengths (t_{on}) and average laser powers (P_{avg}) with the laser power at 600 mW.

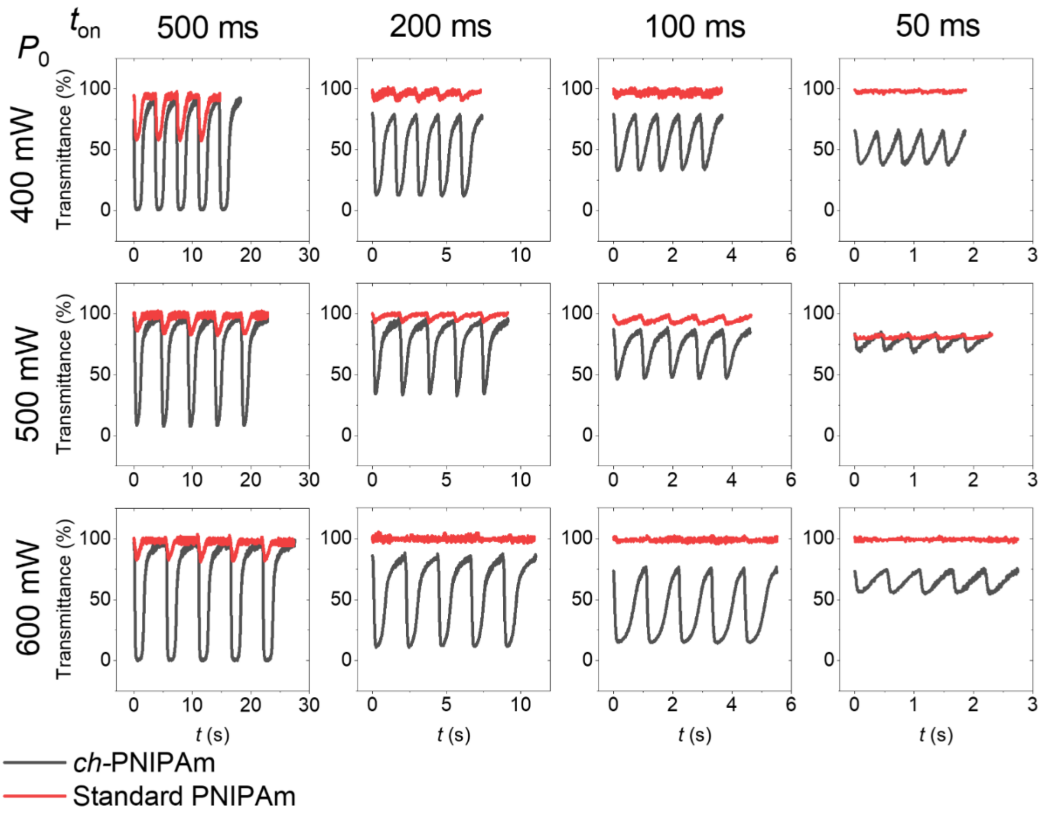

Figure S17. Comparison of the transmittance of *ch*-PNIPAm and standard PNIPAm hydrogel films at different laser pulse lengths (t_{on}) and laser powers (P_0) with the average laser power at 54.5 mW.

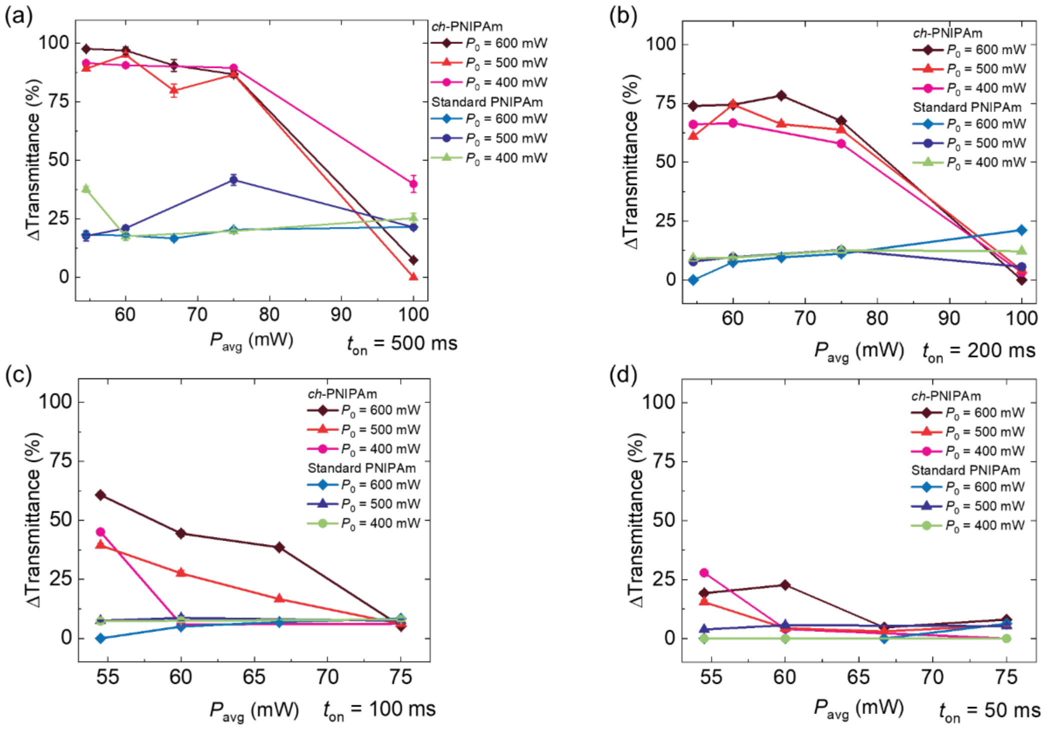

Figure S18. Comparison of the effect of the laser pulse length (t_{on}) on the change of transmittance of *ch*-PNIPAm and standard PNIPAm hydrogel films as a function of the average power (P_{avg}) at different laser powers (P_0). (a) t_{on} = 500 ms, (b) t_{on} = 200 ms, (c) t_{on} = 100 ms, (d) t_{on} = 50 ms. Lines are to guide the eyes.

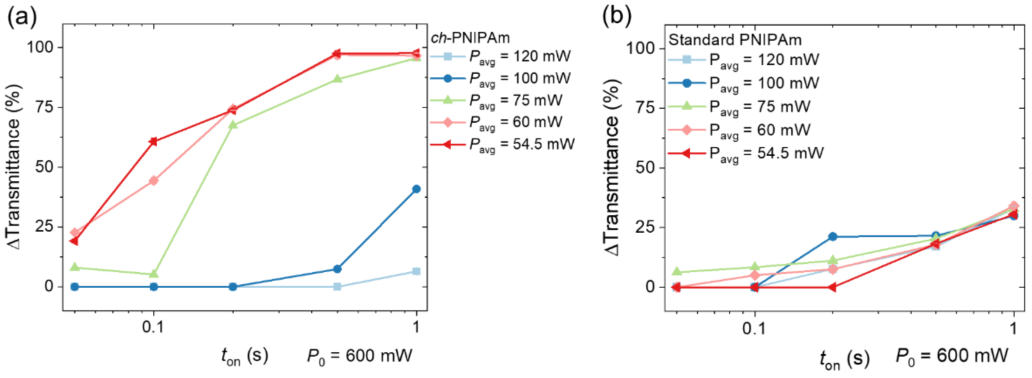

Figure S19. Effect of the average laser power (P_{avg}) on the change of transmittance for (a) *ch*-PNIPAm and (b) standard PNIPAm with the laser power at 600 mW. Lines are to guide the eyes.

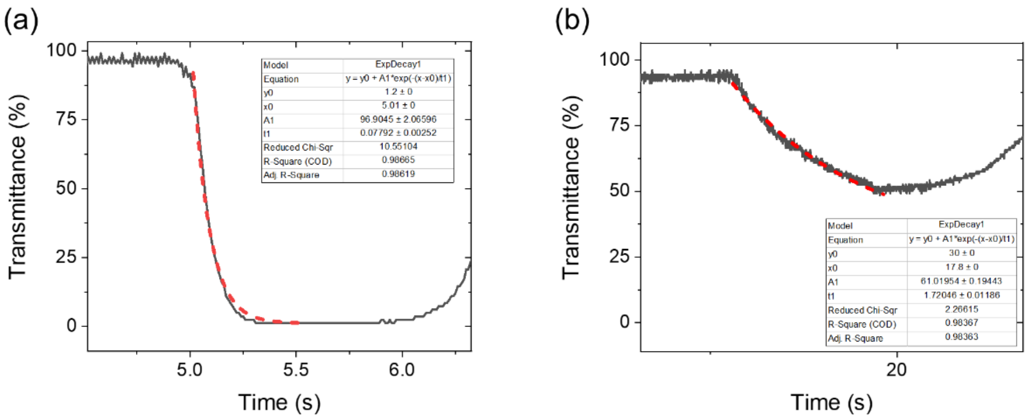

Figure S20. Examples of the exponential fits on the measurement data of the phase transition. Red-dashed lines show the resulting fit. (a) *ch*-PNIPAm hydrogel with t_{on} = 0.5 s, P_{avg} = 60 mW. (b) Standard PNIPAm with t_{on} = 2 s, P_{avg} = 66.7 mW. For both measurements P_0 = 600 mW. The characteristic time τ is denoted as *t1* in the figure.

Caption for Supporting Video S1

Video S1. Reversible switching of the *ch*-PNIPAm projection screen by external heating. A logo image of Aalto University was projected onto the hydrogel film continuously. The hydrogel film inside a capillary was switched on with a heat gun (heating period indicated in the video) to display the projected image. After removing the heat gun, the hydrogel film cooled down and became transparent again, and the image disappeared.
